# Oral Health Across the Menopausal Transition: Biological Pathways, Clinical Implications, and Future Perspectives

**DOI:** 10.3390/jcm15155757

**Published:** 2026-07-23

**Authors:** Andrea Butera, Carolina Maiorani, Andrea Scribante, Ruggero Rodriguez y Baena, Laura Cucinella, Giorgia E. Parrotta, Rossella Elena Nappi

**Affiliations:** 1 Unit of Dental Hygiene, Section of Dentistry, Department of Clinical, Surgical, Diagnostic and Pediatric Sciences, University of Pavia, 27100 Pavia, Italy; andrea.butera@unipv.it (A.B.); andrea.scribante@unipv.it (A.S.); ruggero.rodriguez@unipv.it (R.R.y.B.); 2 SISIO (Italian Society of Oral Hygiene Sciences), 00166 Roma, Italy; 3 Unit of Orthodontics and Pediatric Dentistry, Section of Dentistry, Department of Clinical, Surgical, Diagnostic and Pediatric Sciences, University of Pavia, 27100 Pavia, Italy; 4 Department of Public Health, Experimental and Forensic Medicine, University of Pavia, 27100 Pavia, Italy; laura.cucinella01@universitadipavia.it; 5 Research Center for Reproductive Medicine, Gynecological Endocrinology and Menopause, IRCCS S. Matteo Foundation, 27100 Pavia, Italy; rossella.nappi@unipv.it; 6 Department of Clinical, Surgical, Diagnostic and Pediatric Sciences, University of Pavia, 27100 Pavia, Italy

**Keywords:** menopause, oral health, periodontitis, xerostomia, salivary dysfunction, hormone replacement therapy, oral microbiome, estrogen deficiency

## Abstract

**Background/Objectives:** Menopause is a complex physiological transition characterized by progressive estrogen deficiency and systemic biological changes that can affect multiple organs and tissues, including the oral cavity. Growing evidence suggests that hormonal fluctuations during the menopausal transition may influence periodontal health, salivary function, oral sensory perception, and overall oral health-related quality of life. Objective: This narrative review aims to provide a comprehensive overview of the biological mechanisms and clinical manifestations associated with the relationship between menopause and oral health, with particular attention to periodontal outcomes, salivary changes, oral discomfort, dental status, and the potential role of hormone replacement therapy (HRT). **Methods:** This narrative review was based on a structured literature search conducted in PubMed/MEDLINE and Scopus to identify studies published between January 2005 and May 2026. Predefined eligibility criteria were applied to identify relevant human studies. The retrieved evidence was synthesized narratively according to major oral health domains and menopausal phenotypes. **Results:** Fifty studies met the inclusion criteria. Overall, menopause was associated with poorer periodontal parameters, including increased probing depth, clinical attachment loss, and periodontal inflammation. Reduced salivary flow, dry mouth, altered salivary composition, burning symptoms, and taste disturbances were frequently reported in peri- and postmenopausal women. A higher prevalence of caries and tooth loss was also reported, although the contribution of age and other confounding factors varied across studies. New evidence suggests that estrogen deficiency may influence oral health through interconnected pathways involving immune regulation, bone metabolism, salivary gland function, and host–microbiome interactions. Evidence regarding the effects of HRT has been mixed, although several studies have reported improvements in salivary function and periodontal outcomes among treated women. **Conclusions:** Menopause appears to act as an important systemic modifier of oral health through multifactorial biological mechanisms. Menopause-associated oral manifestations go beyond local tissue changes and reflect broader interactions between hormonal status, inflammation, bone metabolism, and microbial ecology. Increased awareness among dental and medical professionals and a multidisciplinary approach could improve the prevention, diagnosis, and management of oral diseases in postmenopausal women. Further, well-designed longitudinal studies are needed to clarify causal relationships and identify effective therapeutic strategies.

## 1. Introduction

Menopause represents a physiological stage in a woman’s life characterized by the permanent cessation of menstruation resulting from progressive ovarian follicular depletion and the consequent decline in circulating sex hormones, particularly estrogen and progesterone [1,2]. Although traditionally interpreted as the end of reproductive function, menopause is currently recognized as a complex systemic transition associated with biological, metabolic, immunological, and psychosocial changes that may substantially influence women’s overall health and quality of life [3,4].

Increasing evidence suggests that the oral cavity should also be considered among the target sites affected by menopausal hormonal changes. Oral tissues represent a dynamic environment regulated by interactions among epithelial turnover, immune homeostasis, bone metabolism, salivary function, and microbial ecology. Consequently, hormonal fluctuations occurring throughout female life stages—including puberty, pregnancy, and menopause—may directly influence oral physiology and disease susceptibility [4,5].

The biological plausibility of this relationship is supported by the presence of estrogen receptors in the oral mucosa, periodontal tissues, alveolar bone, and salivary glands [2,6]. Estrogen plays an important role in maintaining oral tissue homeostasis; therefore, estrogen deficiency may contribute to increased susceptibility to periodontal inflammation, salivary dysfunction, oral mucosal alterations, and alveolar bone changes [6,7]. Beyond direct tissue regulation, sex steroid hormones appear to play an important modulatory role in oral host–microbiome interactions, influencing epithelial responses, immune signaling, and microbial ecology [7,8].

From a clinical perspective, oral manifestations associated with menopause have attracted increasing attention in recent years. Reviews describe a broad spectrum of oral alterations, including xerostomia, salivary dysfunction, periodontal deterioration, oral mucosal atrophy, burning mouth syndrome (BMS), altered taste perception, increased susceptibility to infections, and impaired oral health-related quality of life (OHRQoL) [1,2,9]. These manifestations may contribute to discomfort, functional limitations, nutritional changes, and reduced psychosocial well-being [1,9].

Recent perspectives emphasize that oral manifestations occurring during menopause should not be interpreted exclusively as local events. Emerging evidence indicates broader interactions among hormonal status, microbiome composition, systemic inflammation, immune regulation, and oral health outcomes [3]. This concept supports a more integrated and multidisciplinary interpretation of menopausal oral health and highlights the importance of collaboration between dental and medical professionals [3,8]. The collection of gut microbial genes involved in estrogen metabolism, known as the estrobolome, has further highlighted the potential interaction between hormonal regulation, microbiome dynamics, and oral health during menopause, although direct evidence linking alterations in the estrobolome to oral outcomes remains limited [3,8].

The relationship between menopause and oral health has been extensively investigated in periodontal research because periodontal tissues appear particularly sensitive to endocrine changes occurring during female aging. Periodontitis is a chronic multifactorial inflammatory disease whose clinical expression depends not only on bacterial biofilm but also on systemic host modifiers [10,11]. Estrogen deficiency has been associated with increased bone turnover, altered inflammatory responses, and periodontal tissue breakdown [7,11,12]. Clinical studies have reported a tendency toward greater probing depth, greater clinical attachment loss, and more severe periodontal conditions in postmenopausal women, although findings remain heterogeneous because of differences in study design and confounding factors [10,13,14].

Hormone replacement therapy (HRT), now more commonly referred to as menopausal hormone therapy (MHT) in several international guidelines, has emerged as a potential modifying factor in periodontal outcomes. Some studies suggest more favorable periodontal conditions among women receiving HRT, although the evidence remains inconsistent, and the underlying mechanisms may involve interactions with vitamin D metabolism and inflammatory regulation [14,15]. Overall, menopause appears to act as a systemic modifier of periodontal health rather than an isolated etiological factor.

Salivary alterations represent another relevant pathway through which menopause may influence oral health. Because salivary glands are hormonally responsive tissues, changes in estrogen availability may affect salivary quantity and composition, contributing to xerostomia and impaired oral homeostasis [2,16,17]. Clinical studies have reported lower salivary flow and suggested possible associations between oral dryness and systemic changes, including reduced bone mineral density, in menopausal women [17,18]. Age-related structural and metabolic modifications of salivary glands may further contribute to oral dysfunction [19].

In addition to salivary dysfunction, menopausal women may experience oral sensory disturbances, including BMS, dysgeusia, and oral discomfort [1,9,17]. Salivary impairment has also been associated with increased caries susceptibility and poorer dental outcomes. A recent meta-analysis reported a higher Decayed, Missing, and Filled Teeth (DMFT) index in postmenopausal women, whereas observational studies suggest that tooth loss may reflect a multifactorial interaction involving endocrine, behavioral, and age-related factors rather than hormonal decline alone [16,20,21]. Collectively, these alterations may negatively affect oral function and OHRQoL [1,17,20].

Menopause is increasingly recognized as a heterogeneous condition encompassing different clinical phenotypes. Perimenopause refers to the transitional period preceding the final menstrual period, whereas postmenopause refers to the period following menopause. Menopause may occur naturally as part of physiological ovarian aging, surgically following bilateral oophorectomy, or be induced by oncologic treatments, including chemotherapy, pelvic radiotherapy, or endocrine therapies that result in estrogen deprivation. These phenotypes differ in the onset and mechanisms of hormonal decline and may therefore have different implications for oral health. Current evidence further suggests that these menopausal phenotypes may influence oral outcomes through distinct biological pathways [4,22,23,24]. In particular, endocrine therapy used to treat hormone-dependent cancers has emerged as a clinically relevant model for investigating hormone-related oral changes, with aromatase inhibitors showing a potential association with poorer periodontal outcomes [23].

Beyond isolated biological alterations, menopause-related hormonal changes may influence oral health through interconnected effects involving inflammatory regulation, salivary characteristics, periodontal status, and women’s perceived physical and emotional well-being, suggesting that oral manifestations should be interpreted within a broader systemic and multidimensional framework [25].

Despite growing scientific interest, the available evidence remains fragmented across oral health domains and heterogeneous with regard to study design, outcome assessment, menopausal classification, and control of confounding variables. Most studies have focused on isolated manifestations—particularly periodontal outcomes or salivary alterations—whereas fewer investigations have adopted an integrated perspective that includes oral symptoms, dental outcomes, oral health-related quality of life, and different menopausal phenotypes. Consequently, a comprehensive synthesis of the biological and clinical dimensions of menopause-associated oral health remains warranted [5,10,23].

Therefore, the aim of the present narrative review is to provide a comprehensive overview of the relationship between menopause and oral health by focusing on the biological mechanisms and clinical manifestations associated with different menopausal phenotypes and by discussing the available evidence regarding periodontal health, salivary alterations, oral discomfort, dental outcomes, oral health-related quality of life, and the implications for multidisciplinary patient management.

## 2. Materials and Methods

### 2.1. Focused Question

The present narrative review aims to provide a comprehensive overview of the relationship between menopause and oral health by evaluating the available evidence regarding the biological mechanisms and clinical manifestations associated with menopausal hormonal changes. Particular attention was given to the impact of menopause on periodontal health, salivary alterations, oral discomfort, dental outcomes, and oral health-related quality of life.

The present manuscript was designed as a narrative review supported by a structured literature search and predefined eligibility criteria to ensure the comprehensive and transparent identification of the available evidence. The review was intended to provide a critical narrative synthesis rather than a systematic evidence synthesis.

### 2.2. Eligibility Criteria

Studies were considered eligible if they investigated the relationship between menopause and oral health outcomes in women undergoing the menopausal transition or in postmenopausal women.

Included studies comprised observational studies, cross-sectional studies, case–control studies, cohort studies, longitudinal studies, and clinical studies published in English. Different menopausal phenotypes, including natural menopause, surgical menopause, oncologic menopause, and treatment-induced hormonal suppression, were considered eligible.

Studies evaluating at least one oral outcome associated with menopause were eligible for inclusion, including:

(i) periodontal health and periodontal disease;
(ii) salivary alterations and xerostomia;
(iii) burning mouth syndrome and oral discomfort;
(iv) dental outcomes, including DMFT index and tooth loss;
(v) oral health-related quality of life.

Studies were excluded according to the following exclusion criteria:

(i) in vitro studies;
(ii) animal studies;
(iii) reviews, including systematic reviews, meta-analyses, scoping reviews, and narrative reviews, because the objective of the present narrative review was to synthesize evidence derived from original studies rather than secondary evidence;
(iv) editorials, letters, and conference abstracts;
(v) studies not published in English; the search was restricted to English-language publications to ensure the consistent evaluation and interpretation of the available evidence;
(vi) studies not focused on menopause or not reporting oral outcomes.

### 2.3. Search Strategy

To support the narrative synthesis, a structured literature search was conducted using the electronic databases PubMed/MEDLINE and Scopus. PubMed/MEDLINE and Scopus were selected because together they provide broad coverage of biomedical, dental, and clinical literature relevant to the objectives of the present review.

Additional studies were identified through manual screening of the reference lists of the included articles.

The search included studies published between January 2005 and May 2026; the final literature search was performed on 10 May 2026. This time window was selected to provide a contemporary overview of the available evidence while reflecting advances in the understanding of menopause-related oral health and current diagnostic and therapeutic approaches.

### 2.4. Search Terms

The search strategy combined controlled vocabulary terms and free-text keywords related to menopause and oral health.

The following combinations were used: (“menopause” OR “perimenopause” OR “postmenopause” OR “menopause iatrogenic” OR “menopausal transition” OR “menopause hormone therapy” OR “adjuvant endocrine therapy” OR “estrogens” OR “androgens” OR “progesterone”) AND (“periodontal disease” OR “tooth loss” OR “dental caries” OR “xerostomia” OR “salivary flow” OR “burning mouth”).

The complete database-specific search strategies are provided in Supplementary Table S1 to ensure the transparency and reproducibility of the literature search.

### 2.5. Screening and Selection of Articles

All records identified through database searching were exported and screened.

In the first phase, titles and abstracts were evaluated according to the eligibility criteria and duplicate records were removed.

In the second phase, the full texts of potentially eligible studies were retrieved and assessed.

Articles that did not meet the inclusion criteria were excluded.

Studies were considered relevant when they specifically investigated the association between menopausal status or menopausal hormonal changes and at least one of the predefined oral health outcomes described in the eligibility criteria.

The final selection included studies investigating the association between menopausal status and oral outcomes.

Screening, study selection, and data extraction were performed by the authors according to the predefined eligibility criteria. Any uncertainties regarding study eligibility or data interpretation were discussed among the authors until consensus was reached.

No review protocol was prospectively registered because the present study was designed as a narrative review supported by a structured literature search rather than as a systematic review.

### 2.6. Data Extraction and Evidence Synthesis

Data extraction was performed manually.

The following information was collected from each included study:

Authors, year of publication, study design, population characteristics, menopausal phenotype, evaluated oral outcomes, exposure variables, and main findings.

Included studies were grouped according to the primary oral outcome investigated to provide a narrative synthesis of the available evidence.

The evidence was organized into the following thematic areas:

(i) periodontal health;
(ii) salivary alterations and xerostomia;
(iii) oral discomfort and burning mouth syndrome;
(iv) dental outcomes;
(v) oral health-related quality of life;
(vi) specific menopausal phenotypes and endocrine-related conditions.

No quantitative synthesis or formal systematic evidence synthesis was planned. Instead, the included studies were critically integrated through a thematic narrative synthesis based on the principal oral health domains.

### 2.7. Quality Assessment of Included Studies

Considering the substantial heterogeneity of the included study designs and outcomes, no single validated risk-of-bias instrument was considered appropriate for all included studies. Therefore, a qualitative methodological appraisal was performed to support the interpretation of the available evidence rather than to determine study eligibility. The methodological quality appraisal was performed by one author according to the predefined qualitative criteria described below. The appraisal considered key methodological domains relevant across observational and clinical studies, including sample selection, exposure assessment, outcome assessment, control of confounding variables, completeness of outcome data (when applicable), and overall methodological consistency.

Each methodological domain was evaluated qualitatively. Studies presenting only minor methodological limitations across most domains were considered to have low methodological concerns, whereas studies showing limitations in several domains were classified as having moderate methodological concerns. Studies presenting substantial methodological limitations affecting multiple domains were classified as having high methodological concerns.

## 3. Results

### 3.1. Study Selection

The study selection process was conducted according to the predefined eligibility criteria.

A total of 5750 records were identified through electronic database searching, including 2297 records retrieved from PubMed/MEDLINE and 3453 records retrieved from Scopus.

Before duplicate removal, the retrieved records were manually inspected and cleaned to remove records considered unsuitable for subsequent screening. After duplicate removal, 1581 records remained and underwent title and abstract screening.

Following the initial screening phase, 1356 articles were excluded because they did not meet the predefined inclusion criteria.

The full texts of 225 potentially relevant studies were subsequently assessed for eligibility.

Studies were excluded at this stage for the following reasons:

(i) lack of relevance to menopause-related oral outcomes;
(ii) ineligible study design;
(iii) non-human populations;
(iv) review articles;
(v) insufficient outcome data.

**Figure 1:**
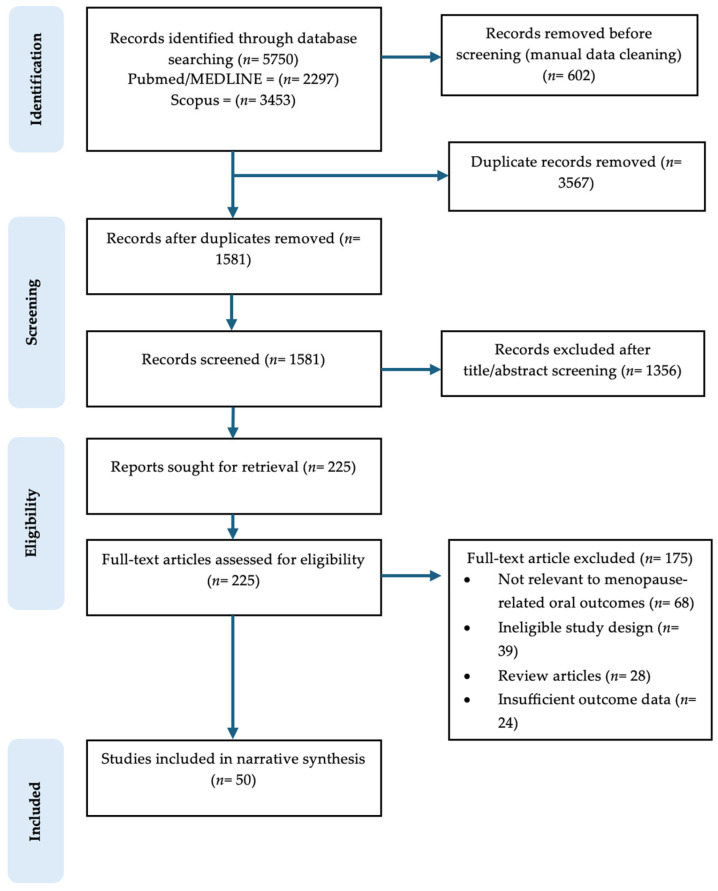
Flow chart of the study.

Ultimately, 50 studies were included in the final narrative synthesis. The selected studies were subsequently synthesized narratively according to the predefined thematic domains (Figure 1).

The review included 50 studies, predominantly observational and cross-sectional studies, with fewer longitudinal, cohort, case–control, and interventional studies. The included studies were conducted across different geographic regions and investigated heterogeneous populations and oral health outcomes.

### 3.2. Characteristics of Included Studies

The included studies were published between 2005 and 2026 and exhibited heterogeneous methodological characteristics.

The evidence comprised observational, cross-sectional, case–control, cohort, longitudinal, and clinical studies investigating oral outcomes associated with menopause.

Study populations included women undergoing the menopausal transition and postmenopausal women, encompassing different menopausal phenotypes such as natural menopause, surgical menopause, oncologic menopause, and treatment-induced hormonal suppression.

The evaluated outcomes covered multiple dimensions of oral health, including periodontal status, salivary alterations and xerostomia, oral discomfort and burning mouth syndrome, dental outcomes including DMFT index and tooth loss, and oral health-related quality of life.

A summary of the characteristics of the included studies is presented in Supplementary Table S2.

#### 3.2.1. Menopause and Periodontal Outcomes

Periodontal outcomes were among the most frequently investigated oral conditions in menopausal women. Overall, several studies reported poorer periodontal parameters in postmenopausal women, although findings were not entirely consistent across populations and study designs.

Higher levels of periodontal inflammation and poorer periodontal parameters were commonly reported in postmenopausal women. Postmenopausal women showed significantly increased plaque index (PI), gingival index (GI), periodontal probing depth (PPD), calculus accumulation, and clinical attachment loss (CAL) compared with premenopausal women [26,27].

Additional evidence supported a greater periodontal burden in postmenopausal populations. Postmenopausal women showed higher CPITN and OHI-S scores and increased prevalence of mobile teeth compared with premenopausal controls [28]. Similarly, an observational study conducted in postmenopausal women attending a dental hospital reported a mean Russell’s periodontal index of 4.34 and an average of 10.3 missing teeth, with most women presenting established or terminal destructive periodontal disease [29].

The duration of menopause also appeared to influence periodontal outcomes. A longer duration of menopause was significantly associated with higher probing pocket depth (*p* = 0.003), greater clinical attachment loss (*p* = 0.009), increased bleeding on probing (*p* = 0.013), and higher plaque index (*p* = 0.010), whereas age itself was mainly associated with tooth loss. Consistently, women with early menopause showed higher CAL, higher HsCRP values, and increased BOP compared with women with menopause at the expected age. Clinical attachment loss was positively associated with menopausal duration and systemic inflammation and negatively associated with estradiol levels [30,31].

Some studies explored biological markers related to periodontal deterioration. Postmenopausal women with chronic periodontitis showed significantly higher serum alkaline phosphatase (ALP), gingival index, probing depth, and clinical attachment loss than postmenopausal women without periodontitis, suggesting a relationship between ALP levels and disease severity [32]. Likewise, estrogen-deficient women with periodontitis showed higher baseline gingival crevicular fluid alkaline phosphatase levels compared with estrogen-sufficient women, although periodontal therapy resulted in reduced inflammatory parameters in both groups during follow-up [33].

However, findings from adjusted analyses were less consistent. Although postmenopausal women were reported to have fewer teeth and slightly greater attachment loss and gingival recession, these associations lost significance after adjustment for confounding variables, including chronological age [13,34]. Similarly, among women already affected by chronic periodontitis, no significant differences in PPD, CAL, plaque accumulation, gingival recession, bleeding indices, or tooth mobility were observed between peri- and postmenopausal women [35].

The effects of HRT were heterogeneous across studies. Postmenopausal women not receiving HRT showed a higher prevalence and risk of periodontitis compared with premenopausal women, whereas periodontal conditions among HRT users were not significantly different from those observed in premenopausal women [30]. Similarly, HRT use was associated with lower odds of periodontal disease after adjustment (OR = 0.79; 95% CI, 0.66–0.94), with stronger associations among women who experienced menopause before the age of 45 years [36].

Interventional studies suggested potential benefits of HRT in women with established periodontal disease. Treatment with estradiol plus dydrogesterone reduced SBI, BOP, IL-6, and TNF-α and improved CAL, alveolar bone height, and bone mineral density over two years [37]. Likewise, adjunctive dydrogesterone combined with periodontal therapy resulted in lower PPD, reduced bleeding indices, lower inflammatory marker concentrations, and favorable changes in oral microbiota compared with periodontal therapy alone [38].

Longitudinal evidence was less conclusive. Some studies observed lower plaque accumulation and gingival bleeding among HRT users but no significant differences in probing depth or clinical attachment loss after adjustment [39], while five-year follow-up data showed only modest periodontal changes and did not support a significant association between hormone therapy and periodontal progression [40].

However, these findings should be interpreted with caution, as most available studies adopted cross-sectional designs and did not fully disentangle the effects of menopausal status from those of chronological aging and other potential confounding factors.

In addition, findings regarding HRT should also be interpreted with caution because women receiving HRT may differ from untreated women with respect to healthcare utilization, health behaviors, and baseline systemic characteristics, potentially influencing the observed associations.

#### 3.2.2. Salivary Outcomes and Xerostomia

Salivary alterations represented one of the most frequently investigated oral health outcomes associated with menopause. Across studies, postmenopausal women generally exhibited reduced salivary secretion, changes in salivary characteristics, and an increased prevalence of xerostomia compared with premenopausal controls [41,42,43,44].

Objective reductions in salivary flow were consistently reported. Postmenopausal women showed significantly lower unstimulated whole salivary flow rates (USFR), with mean values of approximately 0.20 ± 0.11 mL/min compared with 0.40 ± 0.13 mL/min in premenopausal women [43]. Similar reductions in salivary secretion were also observed in studies evaluating stimulated salivary flow [42,45].

Changes in salivary physicochemical properties were also identified. Lower salivary pH, reduced buffering capacity, and increased salivary viscosity were reported among postmenopausal women compared with controls [44,46]. However, not all biochemical salivary parameters appeared altered. One study reported significantly lower salivary flow in postmenopausal women but found no significant differences in salivary pH, phosphate concentration, or dietary phosphorus intake compared with premenopausal women [47].

Subjective xerostomia frequently accompanied objective salivary changes. The prevalence of xerostomia ranged from approximately 57.5% to more than 60% among postmenopausal women and was commonly associated with lower salivary secretion [41,43,44]. In studies evaluating oral discomfort and oral sensory complaints, women with xerostomia also showed lower unstimulated salivary flow and a greater burden of menopausal symptoms [48,49].

Additional analyses explored determinants associated with xerostomia beyond hormonal status alone. Xerostomia was significantly associated with older age, medication use, psychotropic drugs, treatment for climacteric symptoms, frequent nocturnal awakening, headaches, and nasal dryness. However, no significant differences were identified in salivary estradiol concentrations, oral moisture values, or salivary flow between women with and without xerostomia in that population [50].

The effects of HRT on salivary outcomes were heterogeneous. Women receiving HRT showed improved salivary flow, higher salivary pH, and reduced xerostomia-related symptoms compared with untreated postmenopausal women [46]. Another study reported that 72.3% of women receiving HRT had normal salivary flow compared with 38.5% of untreated women, with HRT reducing the probability of low salivary flow (adjusted OR = 0.22) and hyposalivation (adjusted OR = 0.30), while salivary flow increased by approximately 0.52 mL/min. Estro-progestogen regimens showed greater improvements than estrogen-only therapy [51].

Conversely, longitudinal investigations did not consistently confirm a beneficial effect of HRT on salivary secretion. Over a two-year follow-up, no significant differences were observed between HRT users and non-users in resting or stimulated salivary flow, total protein concentration, or salivary IgA levels. However, reductions in salivary albumin, IgG, and IgM concentrations were observed among HRT users during follow-up [52].

#### 3.2.3. Oral Symptoms and Sensory Alterations

Subjective oral symptoms and sensory alterations were frequently reported among peri- and postmenopausal women and represented an additional oral health domain potentially affected during the menopausal transition. Beyond objective salivary alterations, several studies highlighted a high prevalence of oral discomfort and changes in oral perception among menopausal women.

Oral discomfort appeared more prevalent among postmenopausal women than among younger controls. Frequently reported symptoms included burning sensations, altered taste perception, unpleasant oral sensations, and general oral discomfort, often occurring together with xerostomia and reduced salivary secretion [41,53]. Women with oral sensory complaints generally reported a significantly greater burden of menopausal symptoms and lower unstimulated salivary flow rates than women without oral complaints [48].

Burning mouth symptoms were repeatedly reported across studies and represented one of the most frequently investigated oral sensory manifestations associated with menopause. Women reporting burning sensations frequently exhibited concomitant xerostomia and broader alterations in oral sensory perception [48]. In broader evaluations of oral manifestations among postmenopausal populations, BMS was consistently reported together with xerostomia and taste alterations and represented one of the most frequently described oral complaints [53].

Taste disturbances represented another recurrent finding. Altered taste perception was commonly observed during the menopausal transition and frequently coexisted with oral dryness and oral discomfort [48,53]. Some investigations suggested selective impairment of taste perception, whereas others interpreted taste changes as part of a broader oral sensory alteration rather than an isolated gustatory dysfunction [45,48].

Oral sensory complaints were also investigated as combined outcomes. The presence of multiple oral sensory complaints (≥2 OSC), including xerostomia, taste disturbance, and burning symptoms, was associated with significantly greater menopausal symptom burden and lower salivary secretion [48]. These findings suggest that oral sensory symptoms may reflect a broader systemic and menopausal symptom profile rather than isolated oral manifestations.

Factors associated with oral symptoms extended beyond hormonal status alone. Xerostomia and oral discomfort were associated with treatment for climacteric symptoms, medication use, psychotropic drug use, frequent nocturnal awakening, headaches, and nasal dryness, whereas salivary estradiol concentrations and oral moisture values did not significantly differ according to symptom status in some populations [50].

Studies evaluating oral manifestations more broadly also described less frequent mucosal alterations, including oral candidiasis and oral lichen planus, among postmenopausal women, although these findings were considerably less frequent than xerostomia and oral sensory complaints [53].

#### 3.2.4. Dental Outcomes

Dental outcomes were evaluated in several studies through the assessment of dental status, caries experience, tooth retention, oral hygiene indicators, and restorative treatment patterns.

Overall, postmenopausal women showed poorer dental conditions than premenopausal controls, with a higher caries burden and greater tooth loss. Higher DMFT index values were consistently reported after menopause and were frequently accompanied by poorer oral hygiene conditions and greater periodontal treatment needs [42,44].

Higher frequencies of dental caries were also observed among postmenopausal women. Clinical evaluations identified an increased prevalence of carious lesions, together with poorer oral hygiene status, greater plaque accumulation, and increased calculus deposits in menopausal women compared with younger controls [47]. Broader assessments of oral manifestations also described a greater burden of dentition-related deterioration among postmenopausal women [53].

Tooth loss represented another recurrent finding and was consistently more frequent after menopause [29,54]. In one study evaluating estrogen status and bone mineral density, postmenopausal women had significantly more missing teeth than controls regardless of estrogen sufficiency or deficiency, with mean values ranging from approximately 11.8 to 12.5 missing teeth compared with 7.6 teeth in controls [54]. Although women with osteopenia and osteoporosis showed a numerically greater mean number of missing teeth than controls, these differences did not reach statistical significance according to bone mineral density categories [54].

Longitudinal observations suggested that dental deterioration might continue after menopause. Five-year follow-up data indicated that tooth loss occurred more frequently among women with osteoporosis and more severe baseline periodontal disease, although overall periodontal progression remained relatively limited over time [40].

Studies investigating the influence of HRT yielded heterogeneous findings. Longitudinal analyses did not identify significant differences between HRT users and non-users in terms of the DMFT index, number of teeth, periodontal treatment needs, or salivary flow. However, women receiving HRT underwent a greater number of restorative dental procedures during follow-up and reported more frequent dental attendance than untreated women [55].

Beyond caries experience and tooth loss, additional dental findings included poorer oral hygiene scores, increased plaque accumulation, gingival recession, calculus deposition, and greater tooth mobility among postmenopausal women, supporting the presence of a broader deterioration in oral health after menopause [44,47].

#### 3.2.5. Specific Menopausal Phenotypes and Endocrine-Related Conditions

Most of the included studies investigated women with natural postmenopause, whereas fewer studies specifically evaluated perimenopausal women, early menopause, surgical menopause, or endocrine-related conditions [31,38,41,56,57]. Studies conducted in perimenopausal women mainly focused on the effects of progesterone or dydrogesterone on periodontal inflammation, showing context-dependent effects depending on concomitant periodontal therapy [38,56]. Limited evidence also addressed early menopause, suggesting greater periodontal inflammation compared with normal menopause [31], while only a few studies included women with artificial or surgical menopause [41,57]. Overall, direct comparisons among menopausal phenotypes remain limited, and further research is required to clarify phenotype-specific oral health profiles.

#### 3.2.6. Oral Health-Related Quality of Life

Evidence regarding OHRQoL was limited. Only a small number of included studies evaluated patient-reported outcomes related to oral health, generally as secondary rather than primary endpoints [48,50]. In one study, the General Oral Health Assessment Index (GOHAI) was used to assess OHRQoL, whereas the 36-Item Short-Form Health Survey (SF-36) was employed to evaluate general quality of life [48,50]. The included studies also used symptom-specific questionnaires and menopause-related symptom assessment scales to evaluate oral dryness, oral sensory complaints, and menopausal symptoms rather than overall OHRQoL [48,50]. Due to the limited number of available studies and the heterogeneity of the assessment methods, no separate narrative synthesis could be performed. Nevertheless, the available evidence suggests that menopause-related oral symptoms, particularly xerostomia and oral sensory complaints, may negatively influence patients’ perceived oral well-being [48,50].

#### 3.2.7. Summary of the Evidence Across Oral Health Domains

To facilitate the interpretation of the narrative synthesis, the main findings were summarized according to the principal oral health domains, considering the predominant study designs, overall findings, major methodological limitations, and overall interpretation of the available evidence (Table 1).

The interpretation of the evidence presented in Table 1 was also informed by the qualitative methodological appraisal of the included studies (Supplementary Table S3).

HRT: Across the included studies, HRT was evaluated as a potential modifying factor rather than as a separate oral-health domain. Findings were heterogeneous, with some studies reporting favorable periodontal or salivary outcomes and others showing no significant benefit after adjustment or longitudinal follow-up.

### 3.3. Risk of Bias Assessment

The methodological quality of the included studies was evaluated through the assessment of key sources of potential bias based on the information reported in each article. Considering the observational nature and heterogeneity of the included study designs, the assessment focused on five methodological domains: sample selection, exposure assessment, outcome assessment, control of confounding factors, and completeness of outcome data, when applicable. Each methodological domain was qualitatively classified as presenting low, moderate, or high methodological concerns according to the adequacy of methodological reporting and the likelihood of systematic error. An overall methodological quality judgment was subsequently assigned to each study based on the combined evaluation of all domains in Supplementary Table S3.

## 4. Discussion

The present narrative review provides an integrated overview of the available evidence regarding the relationship between menopause and oral health across multiple oral domains, including periodontal conditions, salivary alterations, oral sensory symptoms, dental outcomes, oral health-related quality of life, and different menopausal phenotypes. Overall, the findings support the concept that menopause should not be interpreted as an isolated hormonal event but rather as a systemic transition that may influence oral homeostasis through complex interactions among endocrine regulation, inflammatory pathways, salivary function, microbial ecology, bone metabolism, and behavioral determinants. However, the strength of the available evidence varied across oral health domains. Periodontal outcomes and salivary alterations were supported by the largest and generally most consistent body of evidence, whereas findings regarding oral sensory symptoms and dental outcomes were based on fewer, predominantly observational studies and should therefore be interpreted more cautiously. The interpretation of findings also took into account the methodological quality of the included studies, with greater caution applied to findings derived from studies presenting greater methodological limitations or limited control of potential confounding variables (Supplementary Table S3).

While the included clinical studies consistently described several oral manifestations associated with menopause, many of the biological mechanisms discussed below are derived from the broader scientific literature and are intended to provide a pathophysiological framework for interpreting the available clinical evidence rather than to represent direct findings of the included studies.

Among the investigated outcomes, periodontal alterations and salivary dysfunction emerged as the most consistently reported findings. Postmenopausal women frequently exhibited greater periodontal inflammation, deeper probing depths, higher clinical attachment loss, and poorer periodontal indices compared with premenopausal controls [26,27,29]. However, some studies reported attenuation of these associations after adjustment for confounding factors, highlighting the difficulty of separating the independent contribution of menopausal status from chronological aging [34,54].

Similarly, salivary dysfunction, including reduced salivary flow, increased xerostomia prevalence, and altered salivary characteristics, represented one of the most consistently reported manifestations across studies [41,43,44,46].

Dental outcomes also appeared less favorable after menopause, with a higher caries burden and a greater prevalence of tooth loss reported across different populations [19,20].

The present findings suggest that menopause-associated oral manifestations result from the interaction of multiple biological mechanisms rather than isolated pathological processes. Salivary dysfunction may compromise lubrication, buffering capacity, antimicrobial defense, and oral homeostasis, thereby increasing susceptibility to oral discomfort and caries development. Likewise, inflammatory and tissue remodeling processes occurring in periodontal tissues may contribute to progressive deterioration of oral function and long-term tooth retention. Together, these observations are consistent with the previous literature proposing that oral homeostasis results from dynamic interactions among endocrine regulation, immune responses, microbial mechanisms, and tissue turnover [4,7,8,58,59].

From a biological perspective, estrogen deficiency appears to represent one of the most plausible mechanisms linking menopause and oral alterations. Oral tissues have increasingly been recognized as hormone-responsive structures due to the presence of estrogen receptors in the oral mucosa, periodontal tissues, alveolar bone, and salivary glands [2,6,59]. Estrogen is involved in the regulation of bone metabolism, epithelial turnover, vascular permeability, immune signaling, and salivary gland physiology. Consequently, declining estrogen levels may contribute to increased osteoclastic activity, altered collagen metabolism, enhanced inflammatory responses, and impaired tissue regeneration [2,7,12]. The distribution of estrogen receptors varies across oral tissues. Estrogen receptor beta (ERβ) is extensively expressed in keratinocytes of the oral buccal and gingival epithelia, as well as in acinar and ductal cells of the salivary glands [60]. Both estrogen receptor alpha (ERα) and ERβ are expressed in the periodontal ligament and alveolar bone [61]. This distribution may partly account for the heterogeneous effects of menopause on the outcomes assessed in the reviewed studies.

Recent evidence suggests that menopausal hormonal changes may influence oral homeostasis beyond direct endocrine effects, potentially involving modifications in microbial composition and host–microbiome interactions. Hormonal decline may contribute to changes in salivary characteristics, epithelial responses, immune regulation, and local ecological balance. These changes may, in turn, influence periodontal susceptibility and oral tissue homeostasis [60]. Although direct clinical evidence remains limited, these mechanisms may partly explain the coexistence of multiple oral manifestations observed during the menopausal transition.

The periodontal findings observed in this review appear particularly relevant because periodontitis represents a multifactorial condition in which local microbial stimuli interact with systemic host susceptibility. Several studies reported greater periodontal destruction among postmenopausal women, whereas others reported attenuation of associations after adjustment for confounding variables including age, smoking habits, oral hygiene practices, and socioeconomic conditions [34,36,54]. These findings suggest that menopause alone may not directly determine periodontal disease but may instead interact with existing inflammatory and behavioral risk profiles, potentially contributing to increased susceptibility in selected populations.

Bone-related mechanisms may further contribute to the relationship between menopause and oral outcomes. Reduced estrogen availability has been associated with increased skeletal turnover and lower bone mineral density, potentially influencing alveolar bone support and periodontal stability [12]. However, the available evidence remained heterogeneous. While some studies reported associations between lower bone mineral density and less favorable periodontal conditions, others failed to confirm significant relationships between systemic bone status and periodontal outcomes [23,40,62]. These discrepancies reinforce the concept that periodontal deterioration observed after menopause is likely the result of multiple interacting factors, including endocrine changes, inflammatory responses, behavioral factors, and systemic bone metabolism, rather than isolated endocrine effects.

The influence of HRT also deserves particular attention. Several studies reported more favorable oral conditions among women receiving hormonal treatment, including lower prevalence of xerostomia, improved salivary flow, lower periodontal inflammation, and reduced prevalence of periodontitis [15,36,46,51]. Nevertheless, longitudinal investigations did not consistently demonstrate clinically meaningful improvements in periodontal progression or salivary secretion [40,52,55]. Taken together, these findings suggest that HRT may be associated with favorable outcomes in selected oral domains. However, the available evidence remains heterogeneous and is largely based on observational studies. Therefore, current evidence is insufficient to support specific oral-health recommendations or to consider HRT as a strategy for the prevention or management of oral diseases.

Moreover, decisions regarding HRT should always be individualized according to current clinical guidelines, taking into account the overall risk–benefit profile and potential contraindications, including hormone-sensitive malignancies such as breast and endometrial cancer. Consequently, HRT should not be considered or prescribed for the purpose of improving oral health alone.

Interestingly, the available evidence suggests that the effects of HRT may differ according to the oral health domain investigated. Improvements were more consistently reported for salivary outcomes and xerostomia than for periodontal endpoints. This pattern may suggest that salivary tissues could be more responsive to hormonal modulation than periodontal tissues, although available evidence remains insufficient to establish domain-specific biological effects. Moreover, the impact of HRT may vary according to its components, including both the estrogen component (type, dose, and route of administration) and the progestogen component, which may have different affinities for the progesterone receptor (PgR) and the androgen receptor (AR), both expressed in oral tissues, including gingiva, periodontal ligaments, alveolar bone, and dental structure [63]. Therefore, further studies should explore possible biological effects of different HRT regimens in this setting.

Salivary outcomes showed similarly complex patterns. Although reductions in objective salivary flow were repeatedly observed, subjective xerostomia and measured hyposalivation did not consistently overlap [18,43,50]. This discrepancy suggests that oral dryness perception may depend not only on salivary volume but also on qualitative changes in saliva composition, sensory perception, mucosal sensitivity, and neuroendocrine regulation. These findings support the need for a multidimensional evaluation of oral symptoms in menopausal women rather than relying exclusively on salivary flow measurements.

The present review also highlighted the importance of oral sensory manifestations, including burning symptoms, altered taste perception, and generalized oral discomfort. These symptoms frequently coexisted with xerostomia and appeared to be associated with a greater burden of menopausal symptoms rather than with isolated oral changes [48,50,53]. This observation suggests that oral sensory symptoms may reflect integrated neuroendocrine and mucosal responses occurring during the menopausal transition rather than isolated glandular dysfunction.

Another relevant aspect emerging from the present review concerns the potential interaction between menopause and oral microbial ecology. Increasing evidence suggests that sex steroid hormones may influence microbial composition, epithelial responses, and immune homeostasis, potentially contributing to modifications in oral susceptibility patterns [4,7,8,60]. However, direct evidence linking menopause-associated microbial changes to oral outcomes remains limited. This emerging perspective may help explain why oral manifestations associated with menopause frequently involve multiple clinical domains simultaneously rather than isolated tissue-specific alterations. However, these proposed mechanisms are primarily derived from experimental and mechanistic evidence and were not directly investigated in most of the clinical studies included in this review.

Within this broader biological framework, emerging concepts such as the estrobolome may offer an additional interpretive perspective for understanding menopause-associated oral alterations. The estrobolome refers to the collection of microbial genes involved in estrogen metabolism and enterohepatic estrogen recirculation and has been proposed as a theoretical biological framework through which the gut microbiome may influence systemic estrogen homeostasis [61].

However, none of the studies included in the present review directly evaluated estrobolome-related mechanisms or oral microbiome–estrobolome interactions. Therefore, this concept should currently be interpreted as a theoretical and emerging biological hypothesis that may help explain the observed clinical associations rather than as a mechanism directly demonstrated by the studies included in this review. Accordingly, direct evidence supporting a role of the estrobolome in menopause-associated oral health remains insufficient, and additional translational and clinical studies are required before establishing its clinical relevance and implications for oral health.

Different menopausal phenotypes may influence oral outcomes through distinct biological pathways [4,22,23]. In particular, endocrine therapies used in hormone-dependent cancers may represent clinically informative models because they induce more abrupt and profound estrogen deprivation than natural menopause. Recent evidence suggests that endocrine therapies, particularly aromatase inhibitors, may be associated with poorer periodontal conditions, an increased bleeding tendency, greater alveolar bone deterioration, and poorer patient-reported oral outcomes compared with physiological menopause. In contrast, tamoxifen appears to have a comparatively milder impact on periodontal tissues [23]. These findings suggest that both the intensity and the mode of estrogen deprivation may influence oral manifestations beyond chronological aging alone. However, evidence remains limited and additional studies directly comparing menopausal phenotypes are required.

From a clinical perspective, the findings of this review support the inclusion of oral assessment as part of comprehensive menopausal care. Identification of salivary dysfunction, periodontal deterioration, oral discomfort, and early dental changes may facilitate early recognition, periodic oral-health monitoring, and preventive interventions, thereby contributing to preservation of oral function and quality of life. In clinical practice, this approach should include periodic periodontal screening in peri- and postmenopausal women, assessment of xerostomia and salivary flow when clinically indicated, individualized caries-risk assessment in women with hyposalivation, careful review of medications associated with dry mouth, and appropriate differential diagnosis of burning mouth syndrome after exclusion of local, systemic, pharmacological, and psychosocial causes. Greater collaboration among dental professionals, gynecologists, endocrinologists, and primary care clinicians may improve preventive strategies and increase awareness of oral manifestations associated with menopause. Importantly, oral symptoms should not be automatically attributed to menopause, and appropriate evaluation should exclude local, systemic, pharmacological, and psychosocial causes whenever clinically indicated.

Given the heterogeneity of oral outcomes and menopausal phenotypes, individualized preventive approaches, periodic oral-health monitoring, and early recognition of oral manifestations may represent useful strategies for optimizing long-term oral health during the menopausal transition.

At present, however, the available evidence does not support phenotype-specific clinical protocols, and differences among menopausal phenotypes should currently be regarded primarily as an area for future investigation.

This review presents several strengths. To our knowledge, it represents one of the few narrative syntheses integrating multiple oral domains while simultaneously considering menopause as a heterogeneous condition encompassing natural, surgical, oncologic, and treatment-induced phenotypes. Furthermore, the inclusion of both objective clinical outcomes and patient-reported outcomes allowed a broader interpretation of menopause-associated oral changes. The methodological quality assessment additionally supported interpretation of evidence and contextualization of heterogeneity across included studies. Finally, the discussion of emerging concepts, including host–microbiome interactions, endocrine therapy-related oral changes, and estrobolome-related perspectives, contributed to a more contemporary and clinically relevant interpretation of menopause-associated oral outcomes.

However, several limitations should be acknowledged. Most included studies adopted observational and predominantly cross-sectional designs, limiting causal interpretation. Furthermore, because chronological age is closely related to menopausal status, many of the included observational studies were unable to fully disentangle age-related changes from the independent effects of menopause. Considerable heterogeneity was identified regarding menopausal definitions, oral outcome assessment methods, exposure characterization, confounding adjustment, and periodontal classifications. Moreover, evidence remained limited for surgically induced menopause, oncologic menopause, and endocrine treatment-related conditions.

Additional limitations include possible publication bias and variability in reporting standards across studies. Furthermore, because this review included heterogeneous oral outcomes spanning biological, clinical, and patient-reported dimensions, direct quantitative comparison and formal evidence synthesis were not feasible.

Finally, although a methodological quality assessment was performed, the narrative nature of the review and variability in study design should be considered when interpreting the overall conclusions.

Taken together, these findings highlight the need for further well-designed longitudinal studies to better clarify the biological mechanisms underlying menopause-associated oral changes and to support evidence-based multidisciplinary management.

## 5. Conclusions

This narrative review highlights that menopause should be considered a relevant systemic modifier of oral health rather than an isolated reproductive event. Available evidence suggests that hormonal changes occurring during the menopausal transition may influence oral tissues through complex interactions involving immune regulation, salivary function, bone metabolism, microbial ecology, and behavioral factors.

Among the investigated outcomes, salivary dysfunction, xerostomia, periodontal alterations, and poorer dental outcomes emerged as the most consistently reported findings, although substantial heterogeneity across studies limits definitive causal interpretation. Oral manifestations such as burning mouth syndrome, altered taste perception, and oral discomfort also appear to be clinically relevant but remain incompletely understood.

Current evidence further suggests that menopause should not be regarded as a homogeneous condition, as different menopausal phenotypes, including natural, surgical, oncologic, and treatment-induced menopause, may influence oral outcomes through distinct biological pathways.

Overall, these findings support the integration of oral health into multidisciplinary menopausal care and reinforce the importance of preventive and individualized management strategies. Future well-designed longitudinal studies using standardized oral outcomes and clearer menopausal classifications are needed to better define underlying mechanisms and improve clinical management of women throughout the menopausal transition. Further studies are needed to better clarify the role of menopause in periodontal health while adequately controlling for confounding factors, particularly age-related changes. Additional research is also required to better define the role of HRT, as the current evidence remains insufficient to support specific oral health recommendations regarding its use for the prevention or management of oral diseases in menopausal women.

## Figures and Tables

**Table 1:** Domain-level evidence summary.

	Predominant Study Designs	Overall Findings	Main Methodological Limitations	Overall Interpretation
**Periodontal outcomes**	Mainly cross-sectional and observational studies, with some longitudinal and clinical studies	Most studies reported less favorable periodontal parameters in postmenopausal women, although some associations were attenuated after adjustment for confounding variables.	Predominance of observational designs, heterogeneous periodontal definitions, residual confounding, limited longitudinal evidence.	Overall, periodontal outcomes were the most extensively investigated oral domain and showed generally consistent findings across the included studies.
**Salivary outcomes and xerostomia**	Mainly cross-sectional, case–control and observational studies	Most studies reported reduced salivary flow, altered salivary characteristics and a high prevalence of xerostomia, although objective and subjective findings were not always concordant.	Small sample sizes, heterogeneous salivary assessment methods, variability in xerostomia definitions and inconsistent adjustment for medications and systemic conditions.	Available evidence consistently suggests an association between menopause and salivary dysfunction, although the magnitude of these alterations varied across studies.
**Oral symptoms and sensory alterations**	Mainly observational studies	Burning mouth symptoms, taste alterations and oral discomfort were frequently reported, often together with xerostomia and reduced salivary flow.	Limited number of studies, heterogeneous symptom assessment and predominantly subjective outcomes.	Available evidence is more limited than for periodontal and salivary outcomes and should therefore be interpreted cautiously.
**Dental outcomes**	Mainly cross-sectional and observational studies	Several studies reported higher DMFT index, poorer oral hygiene indices and greater tooth loss among postmenopausal women, although the contribution of aging and other confounding factors varied across studies.	Strong influence of age, oral hygiene, periodontal status, access to dental care and socioeconomic factors.	Available evidence suggests less favorable dental conditions after menopause, although an independent effect of menopause cannot be established.

## Data Availability

No new data were created or analyzed in this study. Data sharing is not applicable to this article.

## Supplementary Materials

The following supporting information can be downloaded at https://www.mdpi.com/article/10.3390/jcm15155757/s1, Table S1. Search terms; Table S2. Characteristic of the included studies [13,14,18,26,27,28,29,30,31,32,33,34,35,36,37,38,39,40,41,42,43,44,45,46,47,48,49,50,51,52,53,54,55,56,57,62,64,65,66,67,68,69,70,71,72,73,74,75,76,77]; Table S3. Methodological Quality Appraisal of Included Studies [13,14,18,26,27,28,29,30,31,32,33,34,35,36,37,38,39,40,41,42,43,44,45,46,47,48,49,50,51,52,53,54,55,56,57,62,64,65,66,67,68,69,70,71,72,73,74,75,76,77].

## Abbreviations

The following abbreviations are used in this manuscript:

ALP	Alkaline Phosphatase
BMD	Bone Mineral Density
BMS	Burning Mouth Syndrome
BOP	Bleeding on Probing
CAL	Clinical Attachment Loss
CPI	Community Periodontal Index
CPITN	Community Periodontal Index of Treatment Needs
CRP	C-Reactive Protein
DG	Dydrogesterone
DMFS	Decayed, Missing, and Filled Surfaces
DMFT	Decayed, Missing, and Filled Teeth
ELISA	Enzyme-Linked Immunosorbent Assay
FSH	Follicle-Stimulating Hormone
GCF	Gingival Crevicular Fluid
GI	Gingival Index
HRT	Hormone Replacement Therapy
HsCRP	High-Sensitivity C-Reactive Protein
HT	Hormone Therapy
IgA	Immunoglobulin A
IgG	Immunoglobulin G
IgM	Immunoglobulin M
IL-1β	Interleukin-1 beta
IL-6	Interleukin-6
IL-8	Interleukin-8
LOA	Loss of Attachment
OHI-S	Simplified Oral Hygiene Index
OHRQoL	Oral Health-Related Quality of Life
PI	Plaque Index
PPD	Periodontal Probing Depth
PT	Periodontal Therapy
PTH	Parathyroid Hormone
SRP	Scaling and Root Planing
TNF-α	Tumor Necrosis Factor-alpha
USFR	Unstimulated Salivary Flow Rate
