# Supplementary material for: Oral Health Across the Menopausal Transition: Biological Pathways, Clinical Implications, and Future Perspectives
Source: J Clin Med. 2026 Jul 23;15(15):5757. doi: 10.3390/jcm15155757 (PMC13466539; doi:10.3390/jcm15155757)
Supplement: Supplementary file 1 [file jcm-15-05757-s001.zip › jcm-4406154-supplementary.pdf]

Table S1. Search terms

Database	Complete search strategy	Limits	Date of search	Records retrieved
PubMed/MEDLINE	"menopause" AND "periodontal disease”	English; January 2005–May 2026	May 2026	300
	"menopause" AND "tooth loss"			0
	"menopause" AND “dental caries"			19
	"menopause" AND "xerostomia"			116
	"menopause" AND "salivary flow"			51
	"menopause" AND "burning mouth"			84
	"perimenopause" AND "periodontal disease”			13
	"perimenopause" AND "tooth loss"			0
	"perimenopause" AND “dental caries"			0
	"perimenopause" AND "xerostomia"			11
	"perimenopause" AND "salivary flow"			4
	"perimenopause" AND "burning mouth"			14
	"postmenopause" AND "periodontal disease”			387
	"postmenopause" AND "tooth loss"			150
	"postmenopause" AND “dental caries"			19
	"postmenopause" AND "xerostomia"			89
	"postmenopause" AND "salivary flow"			38
	"postmenopause"AND "burning mouth"			88
	"iatrogenic menopause" AND "periodontal disease”			0
	"iatrogenic menopause" AND "tooth loss"			0
	"iatrogenic menopause" AND “dental caries"			0
	"iatrogenic menopause" AND "xerostomia"			0
	"iatrogenic menopause" AND "salivary flow"			0
	"iatrogenic menopause"AND "burning mouth"			0
	"menopausal transition" AND "periodontal disease”			7
	"menopausal transition" AND "tooth loss"			3
	"menopausal transition" AND “dental caries"			0
	"menopausal transition" AND "xerostomia"			3
	"menopausal transition" AND "salivary flow"			0
	"menopausal transition"AND "burning mouth"			0
	"menopause hormone therapy" AND "periodontal disease”			57
	"menopause hormone therapy" AND "tooth loss"			19
	"menopause hormone therapy" AND “dental caries"			5
	"menopause hormone therapy" AND "xerostomia"			24
	"menopause hormone therapy" AND "salivary flow"			14
	"menopause hormone therapy" AND "burning mouth"			16
	"adjuvant endocrine therapy" AND "periodontal disease”			2
	"adjuvant endocrine therapy" AND "tooth loss"			3
	"adjuvant endocrine therapy" AND “dental caries"			0
	"adjuvant endocrine therapy" AND "xerostomia"			7
	"adjuvant endocrine therapy" AND "salivary flow"			0
	"adjuvant endocrine therapy"AND "burning mouth"			0
	"estrogens" AND "periodontal disease”			248
	"estrogens" AND "tooth loss"			69
	"estrogens" AND “dental caries"			27
	"estrogens" AND "xerostomia"			78
	"estrogens" AND "salivary flow"			35
	"estrogens""burning mouth"			28
	"androgens" AND "periodontal disease”			57
	"androgens" AND "tooth loss"			14
	"androgens" AND “dental caries"			9
	"androgens" AND "xerostomia"			45
	"androgens" AND "salivary flow"			25
	"androgens"AND "burning mouth"			0
	"progesterone" AND "periodontal disease”			72
	"progesterone" AND "tooth loss"			13
	"progesterone" AND “dental caries"			5
	"progesterone" AND "xerostomia"			8
	"progesterone" AND "salivary flow"			13
	"progesterone" AND "burning mouth"			8
Scopus	"menopause" AND "periodontal disease”	English; January 2005–May 2026	May 2026	217
	"menopause" AND "tooth loss"			124
	"menopause" AND “dental caries"			45
	"menopause" AND "xerostomia"			316
	"menopause" AND "salivary flow"			58
	"menopause" AND "burning mouth"			85
	"perimenopause" AND "periodontal disease”			12
	"perimenopause" AND "tooth loss"			0
	"perimenopause" AND “dental caries"			0
	"perimenopause" AND "xerostomia"			15
	"perimenopause" AND "salivary flow"			2
	"perimenopause" AND "burning mouth"			2
	"postmenopause" AND "periodontal disease”			311
	"postmenopause" AND "tooth loss"			195
	"postmenopause" AND “dental caries"			32
	"postmenopause" AND "xerostomia"			275
	"postmenopause" AND "salivary flow"			28
	"postmenopause"AND "burning mouth"			64
	"iatrogenic menopause" AND "periodontal disease”			0
	"iatrogenic menopause" AND "tooth loss"			0
	"iatrogenic menopause" AND “dental caries"			0
	"iatrogenic menopause" AND "xerostomia"			0
	"iatrogenic menopause" AND "salivary flow"			0
	"iatrogenic menopause"AND "burning mouth"			0
	"menopausal transition" AND "periodontal disease”			0
	"menopausal transition" AND "tooth loss"			0

	"menopausal transition" AND “dental caries"			0
	"menopausal transition" AND "xerostomia"			21
	"menopausal transition" AND "salivary flow"			0
	"menopausal transition"AND "burning mouth"			0
	"menopause hormone therapy" AND "periodontal disease”			37
	"menopause hormone therapy" AND "tooth loss"			26
	"menopause hormone therapy" AND “dental caries"			12
	"menopause hormone therapy" AND "xerostomia"			108
	"menopause hormone therapy" AND "salivary flow"			12
	"menopause hormone therapy" AND "burning mouth"			21
	"adjuvant endocrine therapy" AND "periodontal disease”			0
	"adjuvant endocrine therapy" AND "tooth loss"			0
	"adjuvant endocrine therapy" AND “dental caries"			0
	"adjuvant endocrine therapy" AND "xerostomia"			0
	"adjuvant endocrine therapy" AND "salivary flow"			0
	"adjuvant endocrine therapy"AND "burning mouth"			0
	"estrogens" AND "periodontal disease”			227
	"estrogens" AND "tooth loss"			108
	"estrogens" AND “dental caries"			45
	"estrogens" AND "xerostomia"			326
	"estrogens" AND "salivary flow"			39
	"estrogens" AND "burning mouth"			44
	"androgens" AND "periodontal disease”			37
	"androgens" AND "tooth loss"			18
	"androgens" AND “dental caries"			18
	"androgens" AND "xerostomia"			195
	"androgens" AND "salivary flow"			12
	"androgens"AND "burning mouth"			0
	"progesterone" AND "periodontal disease”			111
	"progesterone" AND "tooth loss"			50
	"progesterone" AND “dental caries"			18
	"progesterone" AND "xerostomia"			146
	"progesterone" AND "salivary flow"			21
	"progesterone" AND "burning mouth"			20

Table S2. Characteristic of the included studies.

Article	Population	Intervention/Exposure	Comparison	Main Outcome
**Exogenous progesterone short-term affects the periodontal environment in perimenopausal women**<br><br>**Man et al. 2023 [64]**	Perimenopausal women (45–55 years old) with or without periodontitis receiving progesterone treatment for 3 months; comparison groups included age-matched untreated perimenopausal and postmenopausal women. Final sample: 50 participants per group.	Exposure to exogenous progesterone therapy for 3 months and evaluation of its effects on periodontal clinical and inflammatory parameters.	Perimenopausal women treated with progesterone vs untreated women; women with periodontitis vs without periodontitis; perimenopausal vs postmenopausal women.	Progesterone increased GI, PD, IL-6, and TNF-α in women without periodontitis and mainly increased PD in women with periodontitis. Changes were reversible and no significant attachment loss was observed.
**The Impact of Progesterone and Estrogen on the Tooth Mobility**<br><br>**Peruga et al. 2023 [65]**	Sixty healthy women aged 20–30 years**,** with normal BMI and no previous pregnancy. Participants had either regular menstrual cycles (n=30) or had been taking two-phase combined oral contraceptives for at least 4 months (n=30).	Exposure to physiological hormonal fluctuations (progesterone and estrogen) during the menstrual cycle and exposure to combined hormonal contraceptive**s**.	Comparison between women with natural menstrual cycles and women taking oral contraceptives, evaluated across different cycle phases (menstrual, ovulatory, and luteal phases).	Tooth mobility remained within physiological limits in all participants. Women with natural menstrual cycles showed significantly increased tooth mobility during the luteal phase, whereas women taking contraceptives showed stable mobility over time**.** Canines and molars were consistently more stable than other teeth. Long-term hormonal contraception appeared to exert a stabilizing effect on tooth anchorage**.**
**Association of Follicle Stimulating Hormone, Alkaline Phosphatase, and Chronic Periodontitis in Post-Menopause - A Comparative Study**<br><br>**Bordoloi et al. 2025 [32]**	Sixty postmenopausal women (45–57 years): 30 without chronic periodontitis and 30 with chronic periodontitis.	Assessment of serum FSH and ALP levels and periodontal evaluation in postmenopausal women.	Postmenopausal women with chronic periodontitis vs postmenopausal women without chronic periodontitis.	FSH levels did not differ significantly between groups. ALP, GI, PD, and CAL were significantly higher in postmenopausal women with chronic periodontitis, suggesting ALP as a potential marker of periodontal disease severity.
**Dydrogesterone alleviates periodontitis in perimenopausal women undergoing periodontal therapy by decreasing inflammation and mediating oral microbiota**<br><br>**Man et al. 2025 [38]**	Sixty perimenopausal women (42–47 years old) with mild–moderate periodontitis and abnormal uterine bleeding associated with ovulatory dysfunction (AUB-O).	Periodontal therapy (PT) combined with oral dydrogesterone (DG) 10 mg twice/day for 10 days/month over 3 months.	Periodontal therapy alone (PT) vs periodontal therapy plus dydrogesterone (PT + DG).	PT + DG resulted in lower PPD, BI, BOP, and GCF inflammatory markers (CRP, IL-6, TNF-α) than PT alone. DG also modified oral microbiota, reducing pathogenic genera and increasing beneficial bacteria.
**The influence of sex hormones on proinflammatory cytokines in gingiva of periodontally healthy premenopausal women**<br><br>**Markou et al. 2011 [66]**	Eighteen periodontally healthy premenopausal women (19–25 years old) with regular menstrual cycles.	Exposure to physiological hormonal changes during the menstrual cycle, evaluated at ovulation and at progesterone peak.	Ovulation phase vs progesterone peak phase.	No significant changes were observed in PI, BOP, IL-1β, IL-8, or TNF-α. Only IL-6 levels increased significantly at progesterone peak, without clinical periodontal changes.
**The "double-edged" role of progesterone in periodontitis among perimenopausal women undergoing or not undergoing scaling and root planing**<br><br>**Yan et al. 2023 [56]**	One hundred twenty-nine perimenopausal women (45–55 years old) with mild-to-moderate periodontitis.	Progesterone treatment for 6 months and/or scaling and root planing (SRP).	SRP + PG vs SRP alone; PG alone vs no treatment.	SRP improved periodontal and inflammatory parameters. Progesterone increased periodontal inflammation in women without SRP but reduced inflammatory markers in women undergoing SRP; effects were reversible after withdrawal.
**Oral Disorders and Serum Oestrogen Levels in**	70 women aged 40–60 years:<br>• 35 postmenopausal women,<br>• 35 premenopausal women.	Postmenopausal hormonal status<br>Serum oestradiol levels.	Postmenopausal women vs premenopausal women	Chronic periodontitis was the most common oral disorder in postmenopausal women (54.3%). Hyposalivation was more frequent in

**Postmenopausal Women in Lagos, Nigeria**<br><br>**Ojo et al. 2023 [67]**			Oral disorders according to serum oestrogen levels.	postmenopausal than premenopausal women. Although postmenopausal women with periodontitis showed relatively higher estradiol levels, no significant association emerged between oral disorders and serum oestrogen levels.
**Salivary flow, testosterone, and femur bone mineral density in menopausal women with oral dryness feeling.**<br><br>**Agha-Hosseini et al. 2013 [18]**	60 menopausal women:<br>• 30 with xerostomia,<br>• 30 without xerostomia.	Assessment of salivary flow rate, serum and salivary testosterone concentrations, and femur BMD.	Menopausal women with xerostomia vs menopausal women without xerostomia.	Women with xerostomia showed lower unstimulated salivary flow and femur BMD and higher serum and salivary testosterone levels. Low salivary flow was the strongest factor associated with dry mouth perception
**Oral health in postmenopausal Turkish women**<br><br>**Yalcin et al. 2006 [41]**	348 menopausal women aged 44–65 years attending a menopause clinic:<br>• hormone users (HRT),<br>• non-users.<br>Included:<br>• natural menopause,<br>• artificial/surgical menopause.	Menopause<br>Hormone replacement therapy (HRT).	HRT users vs non-users.	Dry mouth was the most frequent oral complaint (63.8%). Oral dryness increased with menopause duration but was significantly lower in women using HRT. HRT users also showed lower DMFT, DMFS, and CPI values, indicating better oral and periodontal health, whereas non-users presented poorer periodontal status and higher caries indices. Overall, menopausal hormonal changes appeared to negatively affect oral health, while HRT showed a potential protective effect.
**Relationship between menopause and periodontal disease: A cross-sectional study in a Portuguese population**<br><br>**Alves et al. 2015 [13]**	102 women with chronic periodontitis and at least 6 teeth:<br>• 68 postmenopausal women (study group),<br>• 34 premenopausal women (control group).	Exposure to menopausal status and assessment of oral and periodontal conditions.	Postmenopausal vs premenopausal women with chronic periodontitis.	Postmenopausal women had fewer teeth and slightly higher CAL and gingival recession; however, after adjustment for confounders, menopause was not significantly associated with periodontal severity or tooth loss.
**Salivary calcium in postmenopausal women and prevalence of dental caries: A cross sectional study**<br><br>**Shetty et al. 2023 [68]**	80 women:<br>• 40 postmenopausal women with dental caries (case group),<br>• 40 premenopausal women without caries (control group).	Assessment of menopausal status and measurement of unstimulated salivary calcium levels.	Postmenopausal women with caries vs premenopausal women without caries.	Postmenopausal women showed higher salivary calcium levels and markedly higher DMFT scores than controls. No significant correlation was found between salivary calcium concentration and DMFT within groups.
**Qualitative analysis of serum estrogen, parathyroid and calcium levels in postmenopausal women with oral dryness- A case-control study**<br><br>**Singh et al. 2020 [69]**	Sixty postmenopausal women: 30 with oral dryness (case group) and 30 without oral dryness (control group).	Assessment of oral dryness severity and measurement of serum estrogen, serum PTH, and serum calcium levels.	Postmenopausal women with oral dryness vs postmenopausal women without oral dryness.	Women with oral dryness showed lower serum estrogen, higher PTH levels, and higher XI scores (xerostomia scores) than controls. Serum calcium did not differ significantly. Oral dryness severity was inversely correlated with estrogen levels.
**Unstimulated Salivary Estrogen in Postmenopausal Women With and Without Oral Dryness: A Prospective Study**<br><br>**Gupta et al. 2022 [70]**	Seventy postmenopausal women: 35 with oral dryness (case group) and 35 without oral dryness (control group).	Measurement of unstimulated salivary estrogen levels by ELISA; women with low estrogen levels and oral dryness received hormone replacement therapy (HRT) and were reassessed after 3 months.	Postmenopausal women with oral dryness vs postmenopausal women without oral dryness; before HRT vs after HRT in the case group.	Women with oral dryness showed lower salivary estrogen levels than controls. Salivary estrogen increased after HRT and oral dryness symptoms improved after treatment.
**Correlation of serum oestrogen with salivary calcium in post-menopausal women with and without oral dryness feeling**<br><br>**Singh et al. 2012 [71]**	Sixty postmenopausal women: 30 with oral dryness (case group) and 30 without oral dryness (control group).	Assessment of stimulated salivary calcium concentration and serum oestrogen levels.	Postmenopausal women with oral dryness vs postmenopausal women without oral dryness.	Women with oral dryness showed higher salivary calcium and lower serum oestrogen levels than controls. Salivary calcium and serum oestrogen were inversely correlated.
**Assessment of Oral Manifestations, Periodontal Status, and Vitamin D Levels in Postmenopausal Women: A Cross-sectional Study**<br><br>**John 2022 [72]**	Seventy systemically healthy postmenopausal women aged 50–65 years with at least 20 natural teeth.	Assessment of oral symptoms, periodontal examination, unstimulated salivary flow rate, and measurement of salivary vitamin D levels.	Comparison according to salivary vitamin D levels and their association with periodontal status.	Xerostomia, burning mouth, taste alterations, and hyposalivation were frequent. Most participants had insufficient vitamin D levels, which showed a significant negative correlation with periodontal parameters.
**The influence of hormone replacement therapy on the salivary flow of post-menopausal women**<br><br>**Lago et al. 2015 [51]**	86 postmenopausal women:<br>• 47 receiving HRT (case group)<br>• 39 not receiving HRT (control group).	Hormone replacement therapy (HRT):<br>• estrogen alone,<br>• estrogen + progestogen (estroprogestative therapy).	HRT users vs non-users<br>Estrogen therapy vs estroprogestative therapy.	Women receiving HRT showed significantly higher salivary flow than non-users. Normal salivary flow was observed in 72.3% of HRT users versus 38.5% of controls. HRT reduced the risk of low salivary flow by about 78% and hyposalivation by about 70%, with an average increase in salivary flow of 0.52 mL/min. Estroprogestative therapy appeared more effective than estrogen-only therapy in improving salivary secretion.
**Periodontal Disease in Premenopausal and Postmenopausal Nigerian Women: A Comparative Cross-Sectional Study**<br><br>**Ojo et al. 2026 [28]**	70 women aged 40–60 years:<br>• 35 premenopausal women<br>• 35 postmenopausal women.	Exposure to menopausal status and assessment of periodontal and oral health parameters.	Premenopausal women vs postmenopausal women.	Postmenopausal women showed higher CPITN and OHI-S scores, lower unstimulated salivary flow, and a higher prevalence of mobile teeth, indicating poorer periodontal status than premenopausal women.
**The Relationship between Tooth Loss and Gingival Status with Oral Hygiene in Postmenopausal Women**<br><br>**Wulandari et al. 2021 [73]**	One hundred forty-three postmenopausal women (45–59 years old) with periodontitis from Jakarta, Indonesia.	Assessment of oral hygiene, tooth loss, and gingival status in postmenopausal women.	Comparison according to oral hygiene status and association with tooth loss and gingival parameters (cross-sectional analysis).	Tooth loss was not significantly associated with oral hygiene. Higher plaque index and papillary bleeding index were significantly associated with poorer oral hygiene in postmenopausal women.
**Assessment of periodontal status in pre-And postmenopausal women with**	Sixty women aged 40–60 years: 30 premenopausal women and 30 postmenopausal women, with/without chronic periodontitis	Exposure to menopausal status and periodontal clinical assessment.	Premenopausal women vs postmenopausal women.	Postmenopausal women showed higher PI, GI, calculus index, PD, and CAL scores than premenopausal women, indicating greater susceptibility to periodontitis.

**chronic periodontitis: A cross-sectional study**<br><br>**Agrawal et al. 2021 [26]**				
**Is menopause associated with an increased risk of tooth loss in patients with periodontitis?**<br><br>**Alves et al. 2013 [34]**	One hundred and two women (35–80 years old) with chronic periodontitis and at least six teeth: 68 postmenopausal women and 34 premenopausal women.	Exposure to menopausal status and assessment of oral and periodontal characteristics.	Postmenopausal women vs premenopausal women with chronic periodontitis.	Postmenopausal women had fewer teeth than premenopausal women, but after adjustment for age the difference was not significant. No significant differences were observed in the main periodontal parameters between groups.
**Association among menopause, hormone replacement therapy, and periodontal attachment loss in southern Brazilian women**<br><br>**Haas et al. 2009 [30]**	Three hundred twenty-eight Brazilian women aged 40–69 years: premenopausal women, postmenopausal women using HRT (HRT+), and postmenopausal women not using HRT (HRT−).	Exposure to menopausal status and hormone replacement therapy; full-mouth periodontal examination.	Premenopausal women vs postmenopausal HRT− women vs postmenopausal HRT+ women.	Postmenopausal women not using HRT showed a higher prevalence and risk of periodontitis than premenopausal women. Periodontal status in HRT users was similar to premenopausal women, while tooth loss differences were not significant after adjustment.
**Evaluation of efficacy of estrogen and phytotherapy in oral cavity alterations of women in menopause**<br><br>**Giuca et al. 2009 [74]**	Ninety-five menopausal women: 14 untreated controls and 81 receiving therapy (38 estrogen therapy and 43 phytotherapy).	Hormone replacement therapy using estrogen therapy or phytotherapy.	Estrogen therapy vs phytotherapy vs no hormone replacement therapy (control group).	Women receiving treatment showed improvement or resolution of oral symptoms. Estrogen therapy was more effective than phytotherapy in improving salivary changes, whereas gingivitis, bleeding, and taste alterations improved similarly with both treatments.
**Periodontal disease-associated micro-organisms in peri-menopausal and post-menopausal women using or not using hormone replacement therapy. A two-year follow-up study**<br><br>**Tarkkila et al. 2010 [75]**	One hundred thirty-five peri- and postmenopausal women (50–58 years old): HRT users and non-HRT users followed for 2 years.	Use of hormone replacement therapy (HRT) and longitudinal assessment of subgingival periodontal microbiota.	Women using HRT vs women not using HRT over a 2-year follow-up period.	HRT did not significantly change clinical periodontal status but was associated with reduced detection of periodontal pathogens, particularly P. gingivalis, T. forsythia, and P. intermedia during follow-up.
**Assessment of periodontal health status in postmenopausal women visiting dental hospital from in and around Meerut city: Cross-sectional observational study**<br><br>**Deepa et al. 2016 [29]**	Ninety postmenopausal women aged 45–80 years attending a dental hospital in and around Meerut city.	Exposure to postmenopausal status and clinical periodontal examination.	No external comparison group (cross-sectional observational study). Association assessed within postmenopausal women.	Mean missing teeth was 10.3 and mean Russell's periodontal index was 4.34. Most women presented established or terminal destructive periodontal disease, suggesting increased periodontal vulnerability after menopause.
**Hormone replacement therapy and periodontitis progression in postmenopausal women: A prospective cohort study**<br><br>**Man et al. 2024 [37]**	One hundred ninety-four postmenopausal women (50–55 years): 97 receiving HRT (47 with stage II periodontitis, 50 without periodontitis) and 97 controls without HRT.	Hormone replacement therapy with estradiol plus dydrogesterone for 2 years.	HRT vs no HRT; postmenopausal women with stage II periodontitis vs without periodontitis.	In women without periodontitis, HRT did not significantly affect periodontal parameters. In stage II periodontitis, HRT reduced SBI, BOP, IL-6, and TNF-α within 1–2 years and improved CAL, alveolar bone height, and BMD after 2 years, reducing periodontal progression.
**Association of periodontal inflammation, systemic inflammation, and duration of menopausal years in postmenopausal women**<br><br>**Sharma et al. 2018 [31]**	One hundred three postmenopausal women with chronic periodontitis: 53 women with normal menopause and 50 women with early menopause.	Exposure to menopausal duration and evaluation of periodontal status, serum high-sensitivity C-reactive protein (HsCRP), and serum estradiol levels.	Early postmenopausal women vs normal postmenopausal women.	Women with early menopause showed higher CAL, HsCRP, and BOP than women with normal menopause. CAL was positively associated with HsCRP and duration of menopause and negatively associated with estradiol levels.
**Effect of hormone replacement therapy (HRT) on periodontal status of postmenopausal women**<br><br>**Pizzo et al. 2011 [39]**	Ninety-one postmenopausal women: 52 receiving HRT (HRT+) and 39 not receiving HRT (HRT−).	Long-term hormone replacement therapy (≥ 5 years).	Postmenopausal women receiving HRT vs postmenopausal women not receiving HRT.	HRT users showed lower plaque accumulation and lower BOP initially, but after adjustment for plaque levels, differences in gingival bleeding were no longer significant. No significant differences were observed for PD or CAL, suggesting no relevant protective effect of long-term HRT on periodontitis.
**Gingival crevicular fluid alkaline phosphatase levels in postmenopausal women: effects of phase I periodontal treatment**<br><br>**Daltaban et al. 2006 [33]**	Seventy-three postmenopausal women: 36 estrogen-sufficient women receiving estrogen supplementation and 37 estrogen-deficient women not receiving estrogen therapy. Each group was divided into healthy controls and chronic periodontitis subgroups.	Estrogen status (estrogen supplementation vs no supplementation) and Phase I periodontal treatment (oral hygiene instruction, scaling, and root planing) with 1-year follow-up	Estrogen-sufficient periodontitis (ES/P) vs estrogen-deficient periodontitis (ED/P); baseline vs 1 year after periodontal treatment; healthy controls vs periodontitis groups.	Periodontitis groups showed higher PI, GI, BOP, PD, CAL, GCF volume, and GCF ALP than controls. Estrogen-deficient women with periodontitis had significantly higher baseline GCF ALP levels than estrogen-sufficient women. After Phase I therapy, GCF volume and ALP significantly decreased in both periodontitis groups, although ALP remained numerically higher in estrogen-deficient women.
**Menopausal symptoms are associated with oral sensory complaints in perimenopausal women: an observational study**<br><br>**Taga et al. 2021 [48]**	Forty-three perimenopausal women aged 45–55 years recruited from oral medicine and dental hospital settings in Japan.	Assessment of menopausal symptoms, oral sensory complaints, salivary flow, tongue coating, medication use, and quality of life (observational study; no intervention).	Women with vs without xerostomia, taste disturbance, burning mouth, and ≥2 oral sensory complaints (2OSC).	Women with oral sensory complaints showed a higher number of menopausal symptoms and lower unstimulated salivary flow. Logistic regression identified the number of menopausal symptoms as significantly associated with xerostomia, taste disturbance, burning mouth, and multiple oral sensory complaints.
**Factors associated with xerostomia in perimenopausal women**<br><br>**Shinohara et al. 2021 [50]**	One hundred eighteen perimenopausal women (45–55 years old) attending a gynecology outpatient clinic in Japan.	Assessment of menopausal status, oral symptoms, treatment for climacteric symptoms, salivary biomarkers, salivary flow, oral moisture, and quality of life (observational study; no intervention).	Women with xerostomia vs women without xerostomia.	Xerostomia was associated with older age, medication use, psychotropic drugs, HRT, climacteric treatment, and several menopausal symptoms. Logistic regression identified treatment for climacteric symptoms, frequent awakening at night, headaches, and nasal dryness as the main factors associated with xerostomia. No significant differences were observed in salivary flow or salivary estradiol levels between groups
**Saliva in perimenopausal and early postmenopausal women. A 2-year follow-up study**	One hundred sixty-one peri- and early postmenopausal women: 106 consistent HT users and 55 non-users followed for 2 years.	Use of hormone therapy (HT) with longitudinal evaluation of salivary flow and salivary biochemical markers.	Women using HT vs women not using HT; baseline vs 2-year follow-up.	No significant differences were observed in salivary flow, total protein, or IgA between groups or over time. In HT users, albumin, IgG, and IgM concentrations significantly decreased during follow-up, suggesting a possible

**Tarkkila et al. 2011 [52]**				improvement in oral epithelial integrity, although HT did not substantially affect salivary secretion.
**Oral health in perimenopausal and early postmenopausal women from baseline to 2 years of follow-up with reference to hormone replacement therapy**<br><br>**Tarkkila et al. 2008 [55]**	One hundred sixty-one peri- and early postmenopausal women: 106 HRT users and 55 non-HRT users followed for 2 years.	Use of hormone replacement therapy (HRT) with longitudinal evaluation of oral and salivary parameters.	Women using HRT vs women not using HRT; baseline vs 2-year follow-up.	No significant differences were found between HRT users and non-users in dental status, periodontal condition, or salivary flow rates. However, HRT users received more dental restorations during follow-up and reported more recent dental visits, suggesting greater health awareness rather than a direct oral benefit of HRT.
**Differences in periodontal severity between perimenopausal and postmenopausal women with chronic periodontitis**<br><br>**Wulandari et al. 2019 [35]**	Sixty-three women aged 45–59 years with chronic periodontitis: 27 perimenopausal and 36 postmenopausal women. Women with oophorectomy, HRT use, diabetes, smoking, or recent periodontal treatment were excluded.	Exposure to menopausal status (perimenopause vs postmenopause) and periodontal clinical examination.	Perimenopausal women vs postmenopausal women with chronic periodontitis.	No significant differences were found between perimenopausal and postmenopausal women in PD, CAL, gingival recession, plaque, calculus, oral hygiene, papilla bleeding, or tooth mobility. Both groups showed comparable periodontal severity despite age differences.
**Evaluation of Periodontal Health Status in Pre and Postmenopausal Women with Periodontitis**<br><br>**Sahney et al. 2025 [27]**	Thirty women aged 25–65 years with chronic periodontitis: 15 premenopausal women and 15 postmenopausal women. Inclusion criteria included probing pocket depth ≥4 mm, clinical attachment loss ≥3 mm, and at least 15 natural teeth.	Exposure to menopausal status and comprehensive periodontal clinical examination.	Premenopausal women with periodontitis vs postmenopausal women with periodontitis.	Postmenopausal women showed significantly higher PI, GI, PPD, and CAL values than premenopausal women (P=0.01). Xerostomia, oral ulcers, furcation involvement, and tooth loss were more frequent in postmenopausal women, suggesting greater periodontal deterioration after menopause.
**Evaluation of salivary flow rate, PH and buffer in pre, post & post menopausal women on HRT**<br><br>**Mahesh et al. 2014 [46]**	Sixty women aged 35–55 years divided into three groups: 20 premenopausal women, 20 postmenopausal women, and 20 postmenopausal women receiving HRT for more than 6 months.	Exposure to menopausal status and hormone replacement therapy (HRT); salivary assessment under standardized conditions.	Premenopausal women vs postmenopausal women vs postmenopausal women receiving HRT.	Postmenopausal women showed reduced salivary flow, lower salivary pH, increased viscosity, and lower buffer capacity than premenopausal women. Women receiving HRT showed improved salivary flow and oral symptoms compared with untreated postmenopausal women, although values did not fully return to premenopausal levels.
**Five-year changes in periodontal disease measures among postmenopausal females: the Buffalo OsteoPerio study**<br><br>**LaMonte et al. 2013 [40]**	1,025 postmenopausal women aged 53–83 years participating in the Buffalo OsteoPerio Study, with baseline and 5-year follow-up oral examinations.	Exposure to postmenopausal status with longitudinal periodontal assessment over 5 years (observational cohort study; no intervention).	Baseline periodontal status vs 5-year follow-up; subgroup comparisons according to hormone therapy use, osteoporosis status, smoking, age, age at menopause, and baseline periodontal severity.	Over 5 years, periodontal changes were generally small and did not indicate marked disease progression. Alveolar bone loss (ACH) showed slight progression, whereas PD and CAL remained relatively stable. Greater bone loss was observed in women with severe baseline periodontitis, osteoporosis, and tooth loss during follow-up. Hormone therapy use was not associated with significant differences in periodontal changes.
**The influence of hormonal replacement therapy on alveolar ridge bone in perimenopuasal women**<br><br>**Bakalckuz et al. 2006 [76]**	Eighty-two peri- and postmenopausal women (mean age 50 ± 4.69 years), divided into three groups: menstruating women without HRT, non-menstruating women without HRT, and women receiving HRT.	Exposure to hormone replacement therapy (HRT) and assessment of alveolar ridge bone density using digital radiography and systemic bone density evaluation.	Women receiving HRT vs menstruating women without HRT vs non-menstruating women without HRT.	No significant association was found between HRT use and alveolar ridge bone density. A significant negative correlation was observed between FSH levels and mandibular alveolar bone density in menstruating women without HRT, whereas no significant correlations between hormone levels, systemic BMD, and alveolar density were found among HRT users.
**Association of bone mineral density with periodontal status in postmenopausal women**<br><br>**Singh et al. 2014 [62]**	Seventy-eight dentate postmenopausal women aged 46–54 years, classified as osteoporotic (n=31), osteopenic (n=25), or normal BMD (n=22).	Assessment of systemic bone mineral density (lumbar spine DXA) and periodontal examination (observational cross-sectional study; no intervention).	Osteoporotic vs osteopenic vs normal BMD groups.	Lower BMD was significantly associated with worse periodontal status. PD, CAL, and ACH showed significant negative correlations with T-score, while tooth loss due to periodontitis was not significantly associated with BMD. Osteoporotic women had significantly greater periodontal destruction than osteopenic and normal groups.
**Evaluation of the Consequences of Menopause on Women with Respect to Salivary Flow Rate, Gustatory Function, and pH**<br><br>**Agrawal et al. 2022 [45]**	One hundred five women divided into three groups: 35 premenopausal women (control), 35 menopausal women, and 35 postmenopausal women.	Exposure to menopausal status and assessment of salivary and gustatory parameters (observational prospective study; no intervention).	Premenopausal vs menopausal vs postmenopausal women.	Postmenopausal women showed reduced salivary flow rate, lower salivary pH, and significantly reduced sweet taste intensity compared with premenopausal women. No relevant differences were observed for sour, salty, or bitter taste perception.
**Oral Manifestation in Postmenopausal Women of Maharashtra, Western Region**<br><br>**Muneshwar et al. 2023 [53]**	Ninety postmenopausal women attending the Department of Oral Medicine and Radiology in Maharashtra, India. Women receiving HRT, with surgical menopause, dementia, mental illness, or radiotherapy were excluded.	Exposure to postmenopausal status with detailed oral clinical examination and medical history assessment (cross-sectional observational study; no intervention).	Comparison across age groups: 50–60 years, 61–70 years, and >70 years.	Oral manifestations were reported in 76.6% of women. Xerostomia was the most frequent finding (20.2%), followed by periodontitis (15.9%), altered taste (13%), lichen planus (8.6%), and oral candidiasis (7.2%). Most manifestations occurred in women aged 61–70 years.
**Comparative evaluation of unstimulated whole salivary flow rate and oral symptoms in healthy premenopausal and postmenopausal women - An observational study**<br><br>**Gill et al. 2019 [49]**	Two hundred healthy women: 100 postmenopausal women (44–76 years) and 100 premenopausal women (31–45 years).	Exposure to menopausal status with evaluation of oral symptoms and measurement of unstimulated whole salivary flow rate.	Postmenopausal women vs premenopausal women.	Postmenopausal women reported significantly higher prevalence and intensity of oral discomfort than premenopausal women. Mean USFR was lower in postmenopausal women (0.2 ± 0.11 mL/min) than in premenopausal women (0.4 ± 0.13 mL/min). Lower salivary flow was significantly associated with dry mouth, taste disorders, and burning sensation in postmenopausal women.
**Assessment of effect of menopause on saliva and oral health status**<br><br>**Mishra et al. 2021 [42]**	Eighty women: 40 postmenopausal women (45–60 years; Group I) and 40 controls (21–44 years; Group II).	Exposure to menopausal status with assessment of salivary flow, salivary pH, and oral health parameters (cross-sectional observational study; no intervention).	Postmenopausal women vs control (premenopausal) women.	Postmenopausal women showed significantly more xerostomia and hyposalivation, with lower salivary pH and salivary flow compared with controls. They also had worse oral and periodontal outcomes, including higher OHI-S, DMFT, CPI, and LOA values.
**Oral Conditions, Salivary pH, Flow Rate, Phosphate Level, and Phosphorus Intake of**	One hundred sixty-two women: 68 postmenopausal women (45–65 years, menopausal ≥2 years) and 94	Exposure to menopausal status with assessment of intraoral conditions, unstimulated salivary	Postmenopausal women vs premenopausal women.	Postmenopausal women showed significantly higher prevalence of gingival swelling, discoloration, recession, plaque, calculus, caries, tooth mobility, xerostomia, and burning

**Pre- and Postmenopausal Women**<br><br>**Tjahajawati et al. 2024 [47]**	premenopausal women (21–40 years).	characteristics, and phosphorus intake (cross-sectional observational study; no intervention).		sensation. Salivary flow rate was significantly lower in postmenopausal women, whereas no significant differences were found in salivary pH, salivary phosphate levels, or phosphorus intake.
**Assessing the impact of menopause on salivary flow and xerostomia**<br><br>**Minicucci et al. 2013 [43]**	Sixty women: 30 menopausal women (45–64 years, amenorrhea ≥1 year) and 30 healthy controls (20–44 years) with regular menstrual cycles and no oral disease. Sixteen menopausal women were receiving HRT.	Exposure to menopausal status with subjective and objective assessment of salivary function using questionnaires and salivary flow measurement under non-stimulated and stimulated conditions.	Menopausal women vs control (menarche/premenopausal) women; unstimulated vs stimulated salivary flow conditions.	No significant differences were found in subjective xerostomia symptoms between groups. However, menopausal women showed reduced salivary flow under stimulated conditions (S2 and S3), while unstimulated flow did not differ significantly. HRT was not associated with oral symptoms or salivary flow. Overall, menopause was associated with reduced salivary gland response without clinical dry mouth symptoms.
**Evaluation of the periodontal status in menopause women's visiting dental hospital: An observational study**<br><br>**Chandel et al. 2026 [77]**	Seventy-four menopausal women aged 45–70 years attending a periodontology outpatient clinic. Inclusion criteria: stage III/IV periodontitis, grade B/C, and at least 20 teeth.	Exposure to menopausal duration with clinical periodontal evaluation (observational study; no intervention).	Internal comparison according to duration of menopause and age (correlation analysis; no external control group).	Duration of menopause showed significant positive correlations with periodontal deterioration: higher PPD (p=0.003), CAL (p=0.009), BOP (p=0.013), and PI (p=0.010). Age was significantly associated only with greater tooth loss (p=0.010) and not with periodontal indices. The main causes of tooth loss were grossly decayed teeth, caries, and periodontal disease.
**The relationship between hormone replacement therapy and periodontal disease in postmenopausal women: a cross-sectional study the Korea National Health and Nutrition Examination Survey from 2007 to 2012**<br><br>**Lee et al. 2019 [36]**	5,482 naturally postmenopausal women aged 45–74 years from the Korea National Health and Nutrition Examination Survey (KNHANES, 2007–2012). After propensity score matching, 2,070 women were included (1,035 HRT users and 1,035 non-users).	Exposure to hormone replacement therapy (HRT), defined as use of female HRT for at least one month.	Postmenopausal women receiving HRT (HRT+) vs women not receiving HRT (HRT−); subgroup analysis according to age at menopause (<45 vs ≥45 years).	HRT was associated with a lower likelihood of periodontal disease after adjustment for confounders (OR 0.79; 95% CI 0.66–0.94). The protective association was stronger in women with menopause before age 45 (OR 0.55; 95% CI 0.35–0.87). Higher income and oral care product use were associated with lower periodontal disease prevalence, while smoking increased risk.
**Effect of Menopause on Saliva and Dental Health**<br><br>**Rukmini et al. 2018 [44]**	Eighty healthy women: 40 postmenopausal women (41–60 years; case group) and 40 regularly menstruating women (21–31 years; control group).	Exposure to menopausal status with evaluation of stimulated salivary flow, salivary pH, and oral clinical parameters (cross-sectional observational study; no intervention).	Postmenopausal women vs regularly menstruating (premenopausal) women.	Postmenopausal women showed significantly lower salivary pH and salivary flow (P<0.001). Xerostomia was reported in 57.5% of cases and hyposalivation in 45%. OHI-S, DMFT, CPI, and LOA values were significantly higher in postmenopausal women, indicating poorer oral and periodontal health.
**Hormone-related events and periodontitis in women**<br><br>**Romandini et al. 2020 [57]**	10,273 postmenopausal women representative of approximately 6.1 million South Korean women from the Korea National Health and Nutrition Examination Survey (KNHANES IV–VI).	Exposure to hormone-related events, including endogenous factors (menarche age, menopause age, reproductive life duration, pregnancies, abortions, breastfeeding duration, menopause type) and exogenous factors (oral contraceptive use and hormone replacement therapy).	Comparisons according to different hormone-related event categories and between women with and without periodontitis/severe periodontitis.	Severe periodontitis was associated with longer reproductive life and longer breastfeeding duration. Lower odds of severe periodontitis were observed in women with earlier menopause, artificial menopause, more pregnancies/abortions, and older age at first birth. Oral contraceptive use and HRT were not significantly associated with periodontitis after adjustment.
**Effect of hormone replacement therapy on periodontal health in post-menopausal women in Jeddah, Saudi Arabia**<br><br>**Ageel et al. 2025 [14]**	372 postmenopausal Saudi women (≥45 years old) from healthcare facilities in Jeddah: 186 women with generalized periodontitis (cases) and 186 women with healthy periodontium (controls). Inclusion required at least 12 months post-menopause and ≥8 teeth.	Exposure to hormone replacement therapy (HRT), specifically oral estradiol 1 mg daily (duration 1–9 years).	Postmenopausal women using HRT vs postmenopausal women not using HRT. Comparison was also performed according to periodontal disease presence and severity.	HRT use was associated with a lower prevalence of periodontitis (OR=0.31; 95% CI: 0.11–0.89), and after adjustment HRT users showed approximately six times lower odds of periodontitis (OR=0.17; 95% CI: 0.04–0.81). HRT did not significantly affect periodontitis severity, salivary secretion, or bone loss. Lower CAL was initially observed in HRT users but lost significance after adjustment for confounders.
**Menopause: A risk factor for periodontal disease?**<br><br>**Pallos et al. 2006 [54]**	Forty-six postmenopausal women aged 44–68 years (mean 52.2 ± 4.8) and 15 control women aged 35–54 years (mean 44.7 ± 7.5). Inclusion required at least 10 teeth.	Exposure to menopausal status with assessment of serum estrogen levels (sufficient vs deficient) and bone mineral density (normal, osteopenia, osteoporosis).	Postmenopausal women vs controls; comparison according to estrogen status (sufficient vs deficient) and BMD categories (normal vs osteopenic vs osteoporotic).	Postmenopausal women showed significantly greater CAL and more missing teeth than controls. However, no significant differences in periodontal parameters were found according to estrogen levels or BMD categories. The study did not confirm an association between osteoporosis, estrogen deficiency, and periodontal disease severity in this population.

Table S3. Methodological Quality Appraisal of Included Studies

Article	Sample selection	Exposure assessment	Outcome assessment	Confounding control	Incomplete outcome data	Overall risk of bias
**Exogenous progesterone short-term affects the periodontal environment in perimenopausal women**<br><br>**Man et al. 2023 [64]**	Moderate	Low	Low	Moderate	Low	Moderate
**The Impact of Progesterone and Estrogen on the Tooth Mobility**<br><br>**Peruga et al. 2023 [65]**	Moderate	Low	Low	Moderate	Low	Moderate

**Association of Follicle Stimulating Hormone, Alkaline Phosphatase, and Chronic Periodontitis in Post-Menopause - A Comparative Study**<br><br>**Bordoloi et al. 2025 [32]**	Moderate	Low	Low	Moderate	Low	Moderate
**Dydrogesterone alleviates periodontitis in perimenopausal women undergoing periodontal therapy by decreasing inflammation and mediating oral microbiota**<br><br>**Man et al. 2025 [38]**	Moderate	Low	Low	Moderate	Low	Moderate
**The influence of sex hormones on proinflammatory cytokines in gingiva of periodontally healthy premenopausal women**<br><br>**Markou et al. 2011 [66]**	Moderate	Low	Low	Moderate	Low	Moderate
**The "double-edged" role of progesterone in periodontitis among perimenopausal women undergoing or not undergoing scaling and root planing**<br><br>**Yan et al. 2023 [56]**	Moderate	Low	Low	Moderate	Moderate	Moderate
**Oral Disorders and Serum Oestrogen Levels in Postmenopausal Women in Lagos, Nigeria**<br><br>**Ojo et al. 2023 [67]**	Moderate	Moderate	Low	High	Low	Moderate
**Salivary flow, testosterone, and femur bone mineral density in menopausal women with oral dryness feeling.**<br><br>**Agha-Hosseini et al. 2013 [18]**	Moderate	Low	Low	Moderate	Low	Moderate
**Oral health in postmenopausal Turkish women**<br><br>**Yalcin et al. 2006 [41]**	Low	Low	Low	Moderate	Moderate	Low
**Relationship between menopause and periodontal disease: A cross-sectional study in a Portuguese population**<br><br>**Alves et al. 2015 [13]**	Moderate	Low	Low	Low	Low	Low
**Salivary calcium in postmenopausal women and prevalence of dental caries: A cross sectional study**<br><br>**Shetty et al. 2023 [68]**	Moderate	Low	Low	High	Low	Moderate
**Qualitative analysis of serum estrogen, parathyroid and calcium levels in postmenopausal women with oral dryness- A case-control study**<br><br>**Singh et al. 2020 [69]**	Moderate	Low	Low	Moderate	Low	Moderate
**Unstimulated Salivary Estrogen in Postmenopausal Women With and Without Oral Dryness: A Prospective Study**<br><br>**Gupta et al. 2022 [70]**	Moderate	Low	Low	Moderate	Moderate	Moderate
**Correlation of serum oestrogen with salivary calcium in post-menopausal women with and without oral dryness feeling**<br><br>**Singh et al. 2012 [71]**	Moderate	Low	Low	Moderate	Low	Moderate
**Assessment of Oral Manifestations, Periodontal Status, and Vitamin D Levels in Postmenopausal Women: A Cross-sectional Study**<br><br>**John 2022 [72]**	Moderate	Low	Low	Moderate	Low	Moderate
**The influence of hormone replacement therapy on the salivary flow of post-menopausal women**<br><br>**Lago et al. 2015 [51]**	Moderate	Low	Low	Moderate	Low	Moderate
**Periodontal Disease in Premenopausal and Postmenopausal Nigerian Women: A Comparative Cross-Sectional Study**	Moderate	Low	Low	Moderate	Low	Moderate

**Ojo et al. 2026 [28]**						
**The Relationship between Tooth Loss and Gingival Status with Oral Hygiene in Postmenopausal Women**<br>**Wulandari et al. 2021 [73]**	Moderate	Low	Low	Moderate	Low	Moderate
**Assessment of periodontal status in pre-And postmenopausal women with chronic periodontitis: A cross-sectional study**<br>**Agrawal et al. 2021 [26]**	Moderate	Low	Low	Low	Low	Low
**Is menopause associated with an increased risk of tooth loss in patients with periodontitis?**<br>**Alves et al. 2013 [34]**	Low	Low	Low	Moderate	Low	Low
**Association among menopause, hormone replacement therapy, and periodontal attachment loss in southern Brazilian women**<br>**Haas et al. 2009 [30]**	Moderate	Moderate	Low	Moderate	Moderate	Moderate
**Evaluation of efficacy of estrogen and phytotherapy in oral cavity alterations of women in menopause**<br>**Giuca et al. 2009 [74]**	Moderate	Low	Low	Moderate	Moderate	Moderate
**Periodontal disease-associated micro-organisms in peri-menopausal and post-menopausal women using or not using hormone replacement therapy. A two-year follow-up study**<br>**Tarkkila et al. 2010 [75]**	Moderate	Low	Low	High	Low	Moderate
**Assessment of periodontal health status in postmenopausal women visiting dental hospital from in and around Meerut city: Cross-sectional observational study**<br>**Deepa et al. 2016 [29]**	Moderate	Low	Low	Moderate	Low	Moderate
**Hormone replacement therapy and periodontitis progression in postmenopausal women: A prospective cohort study**<br>**Man et al. 2024 [37]**	Moderate	Low	Low	Moderate	Low	Moderate
**Association of periodontal inflammation, systemic inflammation, and duration of menopausal years in postmenopausal women**<br>**Sharma et al. 2018 [31]**	Moderate	Low	Low	Moderate	Low	Moderate
**Effect of hormone replacement therapy (HRT) on periodontal status of postmenopausal women**<br>**Pizzo et al. 2011 [39]**	Moderate	Low	Low	Moderate	Moderate	Moderate
**Gingival crevicular fluid alkaline phosphatase levels in postmenopausal women: effects of phase I periodontal treatment**<br>**Daltaban et al. 2006 [33]**	Moderate	Low	Low	Moderate	Low	Moderate
**Menopausal symptoms are associated with oral sensory complaints in perimenopausal women: an observational study**<br>**Taga et al. 2021 [48]**	Moderate	Low	Low	Moderate	Low	Moderate
**Factors associated with xerostomia in perimenopausal women**<br>**Shinohara et al. 2021 [50]**	Low	Low	Low	Moderate	Low	Low
**Saliva in perimenopausal and early postmenopausal women. A 2-year follow-up study**<br>**Tarkkila et al. 2011 [52]**	Low	Low	Low	Moderate	Low	Low
**Oral health in perimenopausal and early postmenopausal women from baseline to 2 years of follow-up with reference to hormone replacement therapy**	Moderate	Low	Low	Moderate	Low	Moderate

Tarkkila et al. 2008 [55]						
**Differences in periodontal severity between perimenopausal and postmenopausal women with chronic periodontitis**<br><br>**Wulandari et al. 2019 [35]**	Moderate	Low	Low	Moderate	Low	Moderate
**Evaluation of Periodontal Health Status in Pre and Postmenopausal Women with Periodontitis**<br><br>**Sahney et al. 2025 [27]**	Moderate	Low	Low	Moderate	Low	Moderate
**Evaluation of salivary flow rate, PH and buffer in pre, post & post menopausal women on HRT**<br><br>**Mahesh et al. 2014 [46]**	Low	Low	Low	Low	Low	Low
**Five-year changes in periodontal disease measures among postmenopausal females: the Buffalo OsteoPerio study**<br><br>**LaMonte et al. 2013 [40]**	Moderate	Moderate	Low	Moderate	Low	Moderate
**The influence of hormonal replacement therapy on alveolar ridge bone in perimenopuasal women**<br><br>**Bakalckuz et al. 2006 [76]**	Moderate	Low	Low	Moderate	Low	Moderate
**Association of bone mineral density with periodontal status in postmenopausal women**<br><br>**Singh et al. 2014 [62]**	Moderate	Low	Low	Moderate	Low	Moderate
**Evaluation of the Consequences of Menopause on Women with Respect to Salivary Flow Rate, Gustatory Function, and pH**<br><br>**Agrawal et al. 2022 [45]**	Moderate	Low	Low	High	Low	Moderate
**Oral Manifestation in Postmenopausal Women of Maharashtra, Western Region**<br><br>**Muneshwar et al. 2023 [53]**	Low	Low	Low	Moderate	Low	Low
**Comparative evaluation of unstimulated whole salivary flow rate and oral symptoms in healthy premenopausal and postmenopausal women - An observational study**<br><br>**Gill et al. 2019 [49]**	Moderate	Low	Low	Moderate	Low	Moderate
**Assessment of effect of menopause on saliva and oral health status**<br><br>**Mishra et al. 2021 [42]**	Low	Low	Low	Moderate	Low	Low
**Oral Conditions, Salivary pH, Flow Rate, Phosphate Level, and Phosphorus Intake of Pre- and Postmenopausal Women**<br><br>**Tjahajawati et al. 2024 [47]**	Moderate	Low	Low	Moderate	Low	Moderate
**Assessing the impact of menopause on salivary flow and xerostomia**<br><br>**Minicucci et al. 2013 [43]**	Moderate	Low	Low	Moderate	Low	Moderate
**Evaluation of the periodontal status in menopause women's visiting dental hospital: An observational study**<br><br>**Chandel et al. 2026 [77]**	Low	Low	Low	Low	Low	Low
**The relationship between hormone replacement therapy and periodontal disease in postmenopausal women: a cross-sectional study the Korea National Health and Nutrition Examination Survey from 2007 to 2012**<br><br>**Lee et al. 2019 [36]**	Moderate	Low	Low	Moderate	Low	Moderate
**Effect of Menopause on Saliva and Dental Health**<br><br>**Rukmini et al. 2018 [44]**	Low	Low	Low	Moderate	Low	Low
**Hormone-related events and periodontitis in women**	Moderate	Low	Low	Moderate	Low	Moderate

**Romandini et al. 2020 [57]**						
**Effect of hormone replacement therapy on periodontal health in post-menopausal women in Jeddah, Saudi Arabia**<br><br>**Ageel et al. 2025 [14]**	Moderate	Low	Low	Moderate	Low	Moderate
**Menopause: A risk factor for periodontal disease?**<br><br>**Pallos et al. 2006 [54]**	Moderate	Low	Low	Moderate	Low	Moderate
